# Numerical modeling of subduction and evaluation of Philippine Sea Plate tectonic history along the Nankai Trough

**DOI:** 10.1038/s41598-023-45370-2

**Published:** 2023-10-25

**Authors:** E. J. Moreno, V. C. Manea, M. Manea, S. Yoshioka, N. Suenaga, A. Bayona

**Affiliations:** 1 Research Center for Urban Safety and Security, Kobe University, Kobe, 657-8501 Japan; 2 Computational Geodynamics Laboratory, Centro de Geociencias, Universidad Nacional Autónoma de México, Campus Juriquilla, Querétaro, 76230 México; 3 Department of Planetology, Graduate School of Science, Kobe University, Kobe, 657-8501 Japan; 4 Research Center for Earthquake Hazards, Disaster Prevention Research Institute, Kyoto University, Kyoto, 611-0011 Japan

**Keywords:** Tectonics, Geodynamics, Geophysics

## Abstract

The subduction of the Philippine Sea (PHS) plate along the Nankai Trough in in southwest Japan is a relatively recent process compared with subduction along the Japan Trench in northeast Japan. However, the tectonic evolution of the PHS plate along the Nankai Trough is still controversial and not fully understood. There are several competing hypotheses based on different estimates for the time variations of convergence rate and plate age. Our study employs numerical modelling of subduction in order to evaluate the slab evolution for the last 15 Myr and aims to evaluate each tectonic scenario against the present-day slab geometry along a profile passing through the Shikoku and Chugoku regions. The modelling strategy involves a parameter study where subduction initiation and various subduction parameters are analyzed in terms of subduction geometry evolution. Two-dimensional visco-elasto-plastic numerical simulations of spontaneous bending subduction predict that convergence rate and plate age variations play an important role in the evolution of subduction geometry. Modeling results after 15 Myr of evolution reveal that the tectonic model based on a high convergence rate between ~ 15 Ma and ~ 3 Ma produces a slab geometry that agrees well with the observed present-day slab shape specific for the Shikoku and Chugoku regions.

## Introduction

**Figure 1:**
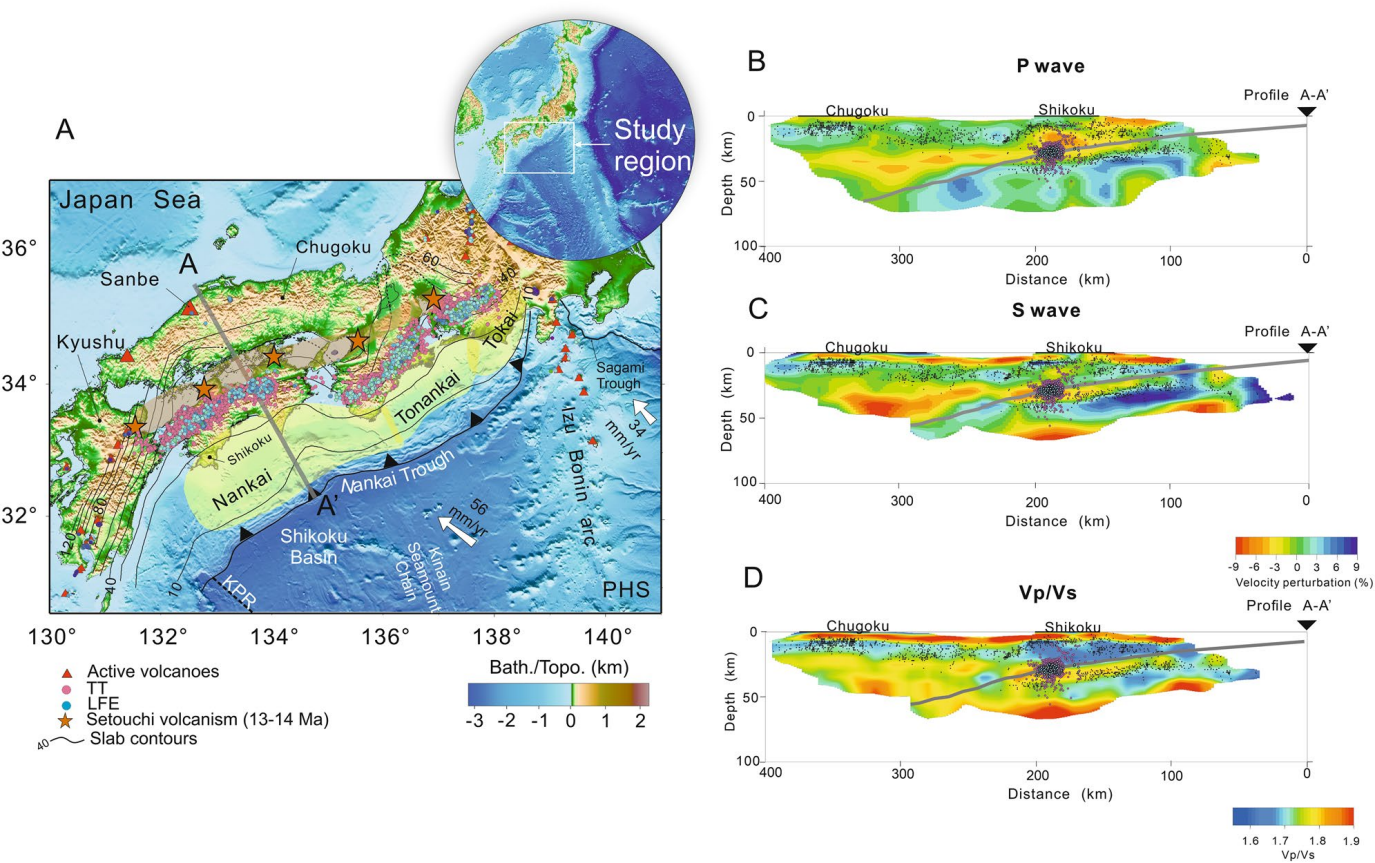
(**A**) Tectonic map with topography and bathymetry of the study area with iso-depths of the upper surface of the PHS slab in SW Japan. Gray thick line A-A’ represents the profile across the Shikoku and Chugoku districts and to the right is the PHS slab geometry^6,10,11^. (**B**) P-wave velocity perturbation with the PHS slab geometry for the profile A-A’ ^12^. (**C**) S-wave velocity perturbation with the PHS slab geometry for the profile A-A’. (**D**) Vp/Vs ratios with the PHS slab geometry for the profile A-A’^12^ taken from https://www.hinet.bosai.go.jp/topics/sokudo_kozo/alljpn_download.php. Black dots in (**B, C**) and (**D**) represent the hypocenters at different depths taken from the JMA unified hypocenter catalogue (https://www.data.jma.go.jp/svd/eqev/data/bulletin/hypo_e.html). The hypocenters are plotted with a width of 15 km to both sides and the data obtained between 2003 and 2020. Red triangles in (**A**) represent active volcanoes. Orange stars represent the Setouchi volcanic arc with ages between 13–14 Ma^3^. White arrows mark the present-day PHS plate motion velocity^13,14^. The green area in (**A**) represents the three Nankai Trough divisions where megathrust earthquakes have occurred historically^10,11,13^. Blue circles in (**A,B,C**) and (**D**) represent low-frequency earthquakes^6–8^ (LFEs) and the data are obtained from the database of JMA and the data obtained between 2003 and 2020 and http://www-solid.eps.s.u-tokyo.ac.jp/~sloweq/?page=map. Pink circles in (**A, B, C**) and (**D**) represent tectonic tremors^14^ (TTs) taken from the database of http://www-solid.eps.s.u-tokyo.ac.jp/~sloweq/?page=map and the data obtained between 2011 and 2015. Other notations: KPR—Kyushu Palau Ridge, PHS—Philippine Sea plate. The gray lines represent PHS slab surface iso-depths with an interval of 10 km, respectively^10^. This figure is created using of GMT 5.3.3 (The Generic Mapping Tools (generic-mapping-tools.org)) and Corel Draw 2022.

The subduction zone along the Nankai Trough experienced different main processes that are related to the expansion of the Japan Sea, and the Southward drift of the SW Japan arc in conjunction with its clockwise rotation^1,2^. However, the current subduction dynamics of the Philippine Sea (PHS) slab is strongly related to its tectonic history and kinematic evolution for the last 15 Myr in SW Japan. The onset of the PHS plate subduction along the Nankai Trough at 15 Ma^1,2^ is still not well understood, and some of the previous main tectonic processes could potentially influenced subduction initiation along this margin^3,4^. Analysis of subduction parameters, as the age and convergence rate of the PHS plate, show a high variability in time and space along the Nankai Trough. For example, the age of the PHS plate was strongly affected by the motion of the Kyushu Palau Ridge (KPR) in the past^3^ (Fig. 1). In terms of plate age along the Nankai Trough, the PHS plate is characterized by two contrasting zones, which are divided by the KPR position^3^. A cold slab covered by a large number of seamounts, near to the Ryukyu Trench with an age of ~ 50 Myr currently subducts beneath the Ryukyu subduction zone in the Kyushu region^3^. In contrast, the Nankai Trough is characterized by the subduction of a young plate with ages between ~ 15 and 25 Ma, which dips into the mantle at angles between ~ 5° and 20° ^3^. Here the overriding plate is characterized by the presence of monogenetic Quaternary volcanoes distantly located from the Nankai Trough (Fig. 1)^3,5,6^. One of the important characteristics of the Nankai subduction zone is the generation of a large number of low-frequency earthquakes (LFEs) and tectonic tremors (TTs) near the trough^3,7^. The presence of LFEs and TTs in the subduction zone may be related to the observed shallow dip angle and dehydration of the young PHS slab ^7–9^ (Fig. 1).

The tectonic evolution of SW Japan is still controversial. However thanks to paleomagnetic reconstructions, the evolution of SW Japan before and after 15 Ma is generally marked by several milestones^1,2,14,15^. Between 30 Ma and 15 Ma Japan was separated from the Asian continent by the Japan Sea rifting^1,3,15–18^. The opening of the Japan Sea caused the clockwise and counterclockwise rotation of the arcs of SW Japan and NE Japan, respectively^1–3,16,17,19^. From ~ 25 Ma to 15 Ma Shikoku Basin is formed as a marginal sea associated with subduction of the Pacific (PAC) plate by rifting located behind the Izu-Bonin-Mariana arc^1,3,4^. The southward drift of the SW Japan in combination with the Japan Sea opening and the clockwise rotation of the SW Japan arc, force the onset of subduction of the Shikoku Basin along the Nankai Trough in SW Japan at 15 Ma^1–3,16,17,19^. The subduction of the young and warm Shikoku Basin subducted beneath SW Japan generated intense volcanism and plutonism, represented by the Setouchi volcanic arc with ages between 14 Ma and 13 Ma^3,20–22^ (Fig. 1). Some studies suggest that subduction must have started with a combination of a low dip angle and high convergence rate due to the young nature of the Shikoku Basin^3,22,23^.

After 15 Ma, SW Japan is not well understood and several hypotheses emerge to try to explain the interaction between the young PHS slab and SW Japan arc from 15 Ma to the present^3,4,19^. In this study, we focus on two relatively contrasting hypotheses: the first assumption proposes that the proto-Izu arc and the Izu-Bonin Mariana Trench reached their present position during the entire Miocene^4^. According to this hypothesis, the PHS slab evolved with an extremely slow convergence rate between ~ 11 Ma and 7 Ma^4^. On the other hand, the second hypothesis is tailored on studies based on the distribution and nature of forearc magmatism in SW Japan between 14 Ma and the present^3^. The volcanism and plutonism between ~ 14–13 Ma suggest the subduction of a young, warm and buoyant plate with a low dip angle^3^. The low pressure and elevated temperature conditions during subduction initiation and short afterwards favored slab melting and consequently, the interaction of the PHS slab with the surrounding mantle produced plutonism and volcanism between ~ 14 Ma and 13 Ma^3,22^ (Fig. 1). In this hypothesis, subduction is self-sustaining and compensates the subducted buoyancy forces of the young Shikoku Basin by initiating subduction at a high convergence rate of ~ 7.3 cm/yr^3^. However, what is the driving force behind the rapid convergence between 15 Ma and 3 Ma is not discussed in this hypothesis. In this tectonic reconstruction, the PHS plate changed its direction of motion from north-northeast to northwest at ~ 3 Ma. After this period, the PHS plate motion velocity decreased to 5.5 cm/yr (until the present) and low dip-angle subduction beneath the Shikoku and Chugoku districts is maintained^3,6,10^*.*

Currently, the Shikoku Basin embracing the PHS plate is subducting with a shallow dip angle, and it is assumed that this type of dynamics is a consequence of the different changes in convergence rates and plate age since 15 Ma^3^. Therefore, our work is focused on understanding under what specific subduction conditions generate a slab shape consistent with the current slab geometry^6,10^ for a 2D profile across Shikoku and Chugoku districts (Fig. 1A). We present a numerical study of subduction based on 2D visco-elasto-plastic formulation that allows us to understand how changes in the convergence rates and plate age proposed by two different hypotheses, namely, Kimura et al. (2014)^4^ and Tatsumi et al. (2020)^3^, affect the PHS plate subduction dynamics. So far, no visco-elasto-plastic modelling of subduction with spontaneous bending have been performed to understand the subduction dynamics of the PHS slab along the Nankai Trough. Our numerical models incorporate temporal variations in convergence rates and oceanic lithospheric thickness with respect to age based on existent studies^3,8^. We investigate how different distributions of plate convergence rate and age affect the subduction dynamics along a 2D profile and compare our predictions in term of slab geometry with the present-day slab shape^6,10^ (Fig. 1B,C,D).

## Slab geometry and volcanism in the Chugoku and Shikoku regions

The PHS slab beneath the Shikoku and Chugoku regions is characterized by low dip-angle subduction where seismicity is observed for depths less than 80 km^6,7,9,10,12^. Actually, the majority of seismicity is concentrated in the first 40 km, where the geometry of the PHS slab is known as shallow subduction^10^ (Fig. 1B,C,D). For the Chugoku region and SW Japan, there are many detailed seismic tomography studies, according to these studies the PHS slab does not reach a depth of 660 km^24–27^. Actually, the Pacific (PAC) slab might act like a barrier so the PHS slab might not reach the 660-km transition zone^10,27^. Moreover, the PSHS slab geometry is identifiable and well-constrained only to shallower depths^10,27^ (Fig. 1B,C,D). Seismic tomographic studies show zones of high velocities beneath the Chugoku region at greater depths in the upper mantle, which are interpreted as the lower end of the PHS slab beneath to Chugoku^6,24^.

The PHS slab beneath the Chugoku district not only has a different subduction style than the PHS slab along the Ryukyu Trench, but also the volcanism in this region differs greatly compared with volcanism present in Kyushu^3,5^. Along the Nankai Trough it is possible to identify different volcanic rocks, as felsic plutonic complexes near to the trough that represent the Setouchi volcanic belt with ages between ~ 14–13 Ma, and Quaternary monogenetic volcanoes such as Sanbe volcano at greater distances from Nankai Trough (Fig. 1A) ^3,5,18,20^. Some studies suggest that the development of magmatic activity in SW Japan may have occurred before 15 Ma, following clockwise rotation and opening of the Japan Sea ^3,5,21,22^. However, after 11 Ma, magmatic activity ceased in this region as a consequence of slow convergence or even the cessation of subduction^4,22^. Other studies propose that the dip angle of the PHS plate has not actually changed significantly during the last 15 Ma^3^. The subduction in the past is characterized by a shallower subduction and the volcanism is originated by mantle wedge melting due to high temperatures of the extremely young PHS lithosphere representative of the Shikoku Basin at 15 Ma^3,22^. Currently, the Sanbe volcano (Fig. 1A) shows traces of adakitic magmas suggesting that the volcanism is mainly due to melting of the lower continental crust^3^ or the interactions of the PHS slab with the upwelling asthenosphere^4,6,26^, and not necessarily by fluids released from the young PHS slab^3,24^.

## Results

### Numerical modelling

It is known that main subduction parameters such as convergence rate and age of the PHS plate may strongly influence on the style of subduction, and subsequently the development of arc volcanism^23,28,29^. Slabs younger than 15 Ma dehydrate at shallower depths and are therefore unable to produce sufficient fluids to generate extensive and well-developed volcanic belts, as in Central America^3,30,31^. At the same time, plate age influences on the slab buoyancy creating favorable conditions for shallow subductions at the onset of subduction^3,23,32^. We construct numerical models based on subduction parameters tailored for a 2D profile specific for the Shikoku and Chugoku regions (Fig. 1A). Our simulations are created using the thermo-mechanical code that solves the equations of mass, momentum and energy for a subduction problem incorporating visco-elasto-plastic rheology (see Methods and Supplementary Information). All simulations presented in this paper are integrated in time 15 Myr. The system of partial differential equations is solved for a 2D Cartesian domain using the finite difference method coupled with marker-in-cell technique for material (rock) transport^33,34^ (Fig. S1). Although seismic tomography studies show the presence of PAC slab approximately 500 km depth below the Shikoku and Chugoku regions^6,10^, our 2D numerical models do not include the subduction dynamics of the Pacific slab from 15 Myr to the present. However, we include a high-viscosity ~ ($1\times {10}^{23}$ Pa s), low temperature layer of ~ 900 °C in our numerical models for depths below 500 km. Assuming it has remained in this position for 15 Myr, this high-viscosity zone approximates the PAC slab located below the PHS slab (Fig. S1). The numerical modelling was performed based on the MATLAB version of the visco-elasto-plastic subduction code presented in Gerya (2019)^33^. We modified the numerical code in order to incorporate time-dependent plate age and convergence rates presented in this study.

In our numerical models, the upper part of the PHS plate is subjected to intense plastic deformation and the lower part of the plate, the deformation is mainly ductile^33,34^. The slab bending is controlled by buoyancy forces and its interaction with the asthenospheric mantle is influenced by the plate motion velocity imposed on the oceanic plate^33,34^. This setting allows to obtain spontaneous bending under both rheological and kinematic conditions^33,34^. Initially, subduction is facilitated by gravitational forces due to density contrast and this is highly dependent on the plate age ^33,34^. In terms of boundary conditions, the right-side lateral boundary of the model domain is time-dependent and controlled by the PHS plate convergence rate specified according to two different hypotheses tested in this work, represented by Model 1 based on Kimura et al. (2014)^4^ and Model 2 based on Tatsumi et al. (2020)^3^, respectively. The bottom model boundary is also displaced in y-direction accordingly in order to satisfy the conservation of mass equation. For subduction initiation we incorporate an inclined mechanically weak zone between the oceanic plate and the overriding plate^33,34^ (Fig. S1). Additionally, we use a variable lithospheric thickness for the oceanic domain that depends on the plate age, while for the continental domain we incorporate a warm region close to the trough (Fig. S1).

**Figure 2:**
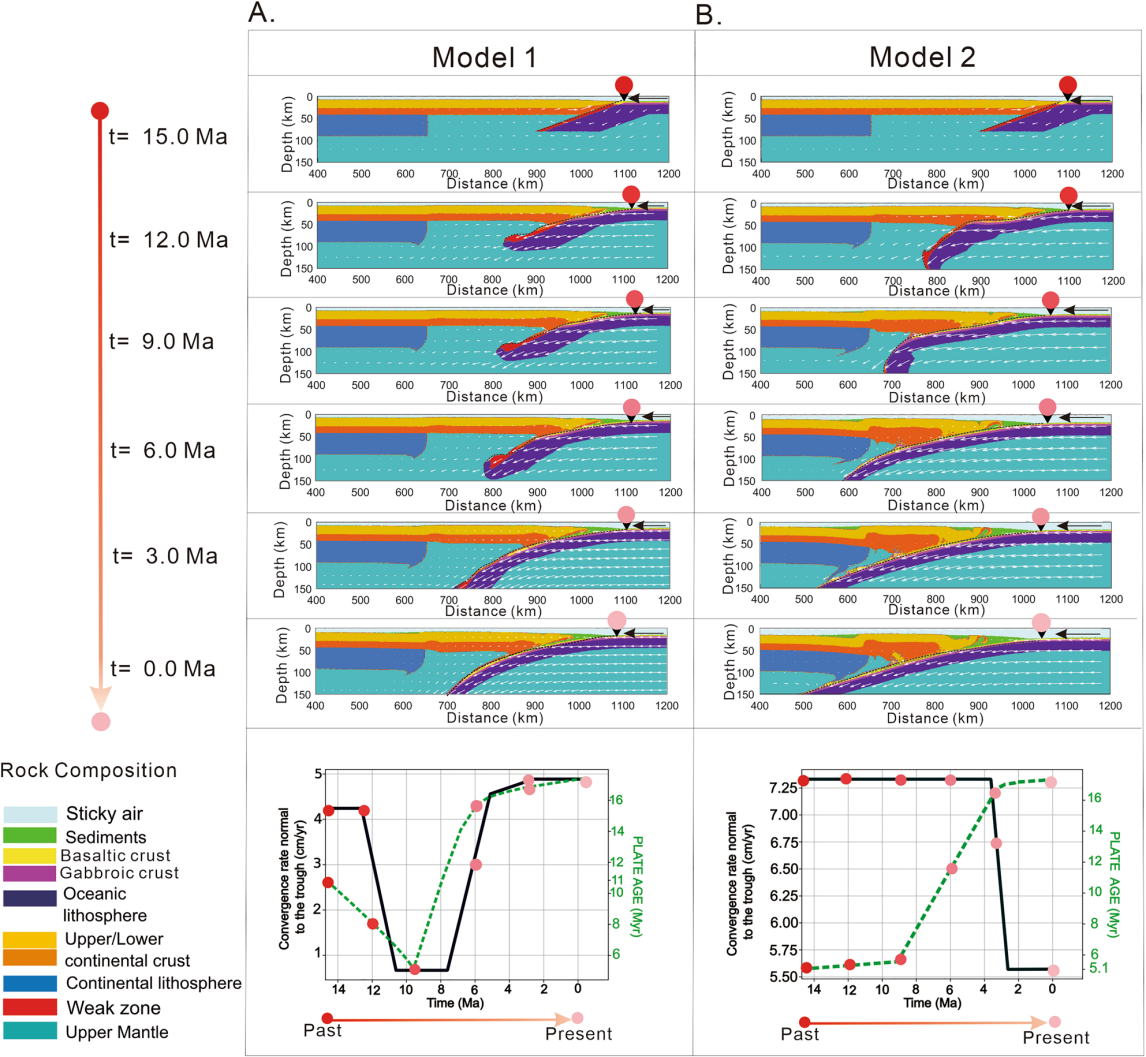
Time series showing modeling results in terms of rock composition for the two tectonic scenarios (Model 1^4^ and Model 2^3^) investigated in this study. Convergence rate and age are shown at the bottom of each time series. The figure shows the time steps each 3 Ma, where the circles that change color from red to pink represent the convergence rate, age, and trough axis position in the time steps indicated. All simulations are performed with an initial dip angle of the weak zone of 20°. (**A**) Model 1 is based on Kimura et al. (2014) ^4^ tectonic model. (**B**) Model 2 is based on Tatsumi et al. (2020)^3^ tectonic model. The black line represents the convergence rate normal at the Nankai Trough, and the green line represents the PHS plate age changes at the trough. This figure is created using Matlab R2022 and Corel Draw 2022.

The convergence rate of the PHS plate with respect to the continental plate during the calculation period is decomposed into two components, one along the study profile and the other along the Nankai Trough axis. The former is used as the convergence rate perpendicular to the Trough axis, and the latter is used to calculate the time variation of the plate age at the starting point of the profile across Chugoku and Shikoku region^8,35^ (Fig. 1A). In all numerical experiments the convergence rate used is the normal component to the trough axis^35^. Temporal plate age at the starting point of the profile is calculated during the period from 15 Ma to the present, using the spatio-temporal distances along to the trough axis between the study profile, the fossil ridge axis, called the Kinan Seamount Chain, and the spreading rate of the Shikoku Basin (Fig. 1)^8,35^. In the first model (Model 1), the PHS plate initiates subduction below Shikoku region with age of ~ 11 Myr and convergence rate of ~ 4.24 cm/yr (Fig. 2A). Model 1^4^ incorporates afterwards a low convergence rate between ~ 11–7 Ma. After 7 Ma convergence rates are no greater than ~ 5 cm/yr (Fig. 2A). The second scenario (Model 2^3^) contains a rather high convergence rate (~ 7.33 cm/yr) between 15–3 Ma, and the PHS plate initiates subduction below Shikoku region with an approximate plate age at the trough of only ~ 5 Myr (Fig. 2B). Numerical results show a high dependence of subduction style on plate ages and convergence rates. Regarding the calculation method for subduction history of the PHS plate, see the Sect. 2.1 in the Supplementary information.

With the addition of the high viscosity zone at the bottom of the model domain, our numerical models show relatively little sensitivity to the prescribed initial dip angle on subduction dynamics (Figs. S2, S3, S5 and S5). Models using convergence rates and plate age specific for Model 1, generate steep dip angle subduction for initial dip angles between 15° and 30° for the weak zone (Figs. S2, S3, S4 and S5). A representative modeling result is shown in Fig. 2A, which is constructed using a prescribed initial weak zone with a dip angle of 20°.

On the other hand, the models constrained using convergence rates and plate ages specific for Model 2^3^, also show a reduced variability in terms of slab geometry. Depending on the initial dip angle (15°–30°), only two subduction styles are obtained from 15 Myr of evolution. These are divided as follows: when the initial dip angle of the weak zone is only 15°, the subduction slab geometry reassembles flat subduction (Fig. S6). Increasing the initial weak zone dip angle (20°–30°), the slab dip gradually increases from a relatively shallow subduction (Figs. S7, S8 and S9). In Fig. 2B we show modeling results using an initial dip angle of 20°, where subduction is characterized by two main stages: flat slab subduction between 15 and 9 Ma, followed by shallow subduction until present day.

## Discussion

### Sensitivity of the slab geometry with respect to plate motion velocity and age parameters

Figure 2 shows the evolution of two subduction models reproduced under different age and plate motion velocity settings using an initial dip angle of the weak zone of 20°. The simulation shown in Fig. 2A considers a relatively low convergence rate followed by a period (11–7 Ma) when the convergence rate drops close to zero and then increases to nearly 5 cm/yr (Fig. 2A). The final result under these conditions is steep dip angle subduction despite the imposition of a low dip angle on the weak zone. This is because the incoming plate age has a distribution that is almost synchronous with the convergence rate (i.e., a low convergence rate corresponds to a young plate age and vice versa).

Figure 2B (Model 2) shows the behavior of the slab under the consideration of a high convergence rate between 15 and 3 Ma, followed by a drop in plate motion velocity. We observe that during 15–9 Ma the plate subducts with a low dip angle (Fig. 2B). This is a consequence of a young and buoyant slab, but as the plate age increases, the subduction angle also increases. In this model the plate age and convergence rate are asynchronous (i.e., a high convergence rate corresponds to a young plate age, and vice versa), and we obtain a slab geometry that corresponds to a rather shallow subduction style. This is consistent with the observation that young slab characterized by periods of high convergence show a predisposition to shallow or even flat subduction, such is the case of the subducted Cocos plate beneath Central Mexico ^36–38^.

One of the controversies about the initiation of subduction along the Nankai Trough is related to the plate motion velocity at which the subduction initiated. There are several mechanisms proposed for the initiation of subduction, which can be induced by transference and polarity reversal due to a passive margin or transform fault gravitational collapse, or even the interaction with a large mantle plume^23,39–41^. In the specific case of the Nankai Trough, several studies argue that this zone was marked by a dextral transform fault and propose that the initiation of subduction is strongly influenced by the opening of the Japan Sea, the separation of SW Japan from the Eurasian plate and the clockwise rotation ~ 45° before 15 Ma^1,2,5,15,16,22^. However, the young PHS plate likely prevents subducting initiation solely by gravitational effects, therefore, the subduction must have been initiated by an accompanying force external to the weight of the slab itself^22,42,43^. Once the subduction process is initiated, a certain mechanism ensures sustaining of subduction for a long period of time^41–43^. This is because buoyancy forces can dominate the system and prevent subduction due to the young age of the slab. A common hypothesis is that subduction initiated at a high convergence rate along a pre-existing weak zone, and despite the young plate age, subduction became self-sustaining^3,15,22,41,43^. Our numerical models support the idea that the PHS plate had to initiate subduction with a high convergence rate and furthermore, this convergence rate had to be maintained for a relatively long period for subduction to prevail, as shown in Fig. 2B.

### Role of the initial dip angle of the weak zone

Since one of the key ingredients related with subduction initiation along the Nankai Trough at 15 Ma is the pre-existence of a weak zone, we test numerical simulation predictions against the initial inclination of the weak zone by varying the dip angle between 15° and 30°. Modelling results show a diversity of subduction styles from steep subduction to shallow subduction and even flat slab subduction, depending on the initial dip angle, convergence rate and age (see Supplementary figures S2–S9).

Incorporating variations tailored to Model 1where the slab velocity comes close to zero between 11 Ma and 7 Ma, we obtained steep dip angle subduction with an initial dip angle of 20° (Fig. 2A, 3A). Using different dip angles for the weak zone (15°–30°) (Figs. S2–S5), the final result in terms of slab geometry is inconsistent with the current slab geometry of the PHS slab beneath Shikoku and Chugoku districts^6,10^ (Fig. 1B).

**Figure 3:**
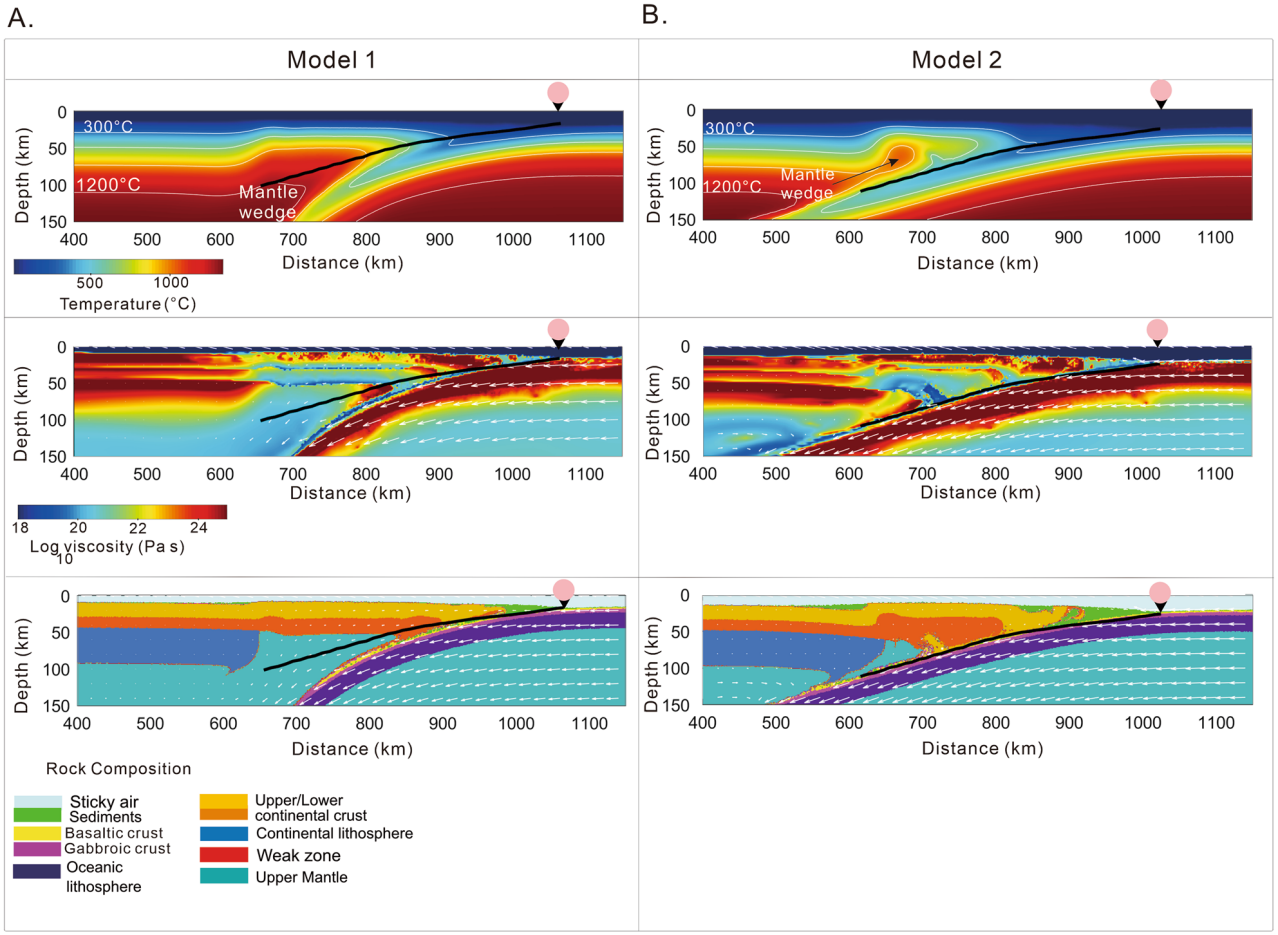
Temperature, viscosity and rock composition distributions after 15 Ma of evolution for the tectonic scenarios presented in Fig. 2 (Model 1, Model 2). (**A**) Model 1 describes steep dip subduction and is not consistent with PHS slab geometry beneath the Chugoku and Shikoku regions^6,10,11^. (**B**) Model 2 shows shallow subduction and high agreement with PHS slab geometry beneath the Chugoku and Shikoku regions^6,10,11^. Solid curves in black represent slab geometry for the 2D profile across the Shikoku and Chugoku regions^6,10,11^. Reversed black triangles with a colored circle on top show the predicted position of the trough. This figure is created using Matlab R2022 and Corel Draw 2022.

However, Model 2 that includes a high convergence rate between 15–3 Ma and initial young plate age (~ 5–6 Myr) (Fig. 2B), we obtained a better fit when an initial dip angle of the weak zone of 20° is used (Fig. 3B). Decreasing more the weak zone dip angle to as low as 15° a flat slab subduction type is obtained (Fig. S6), possible similar to the Chilean or Peruvian flat slabs, but not with the PHS slab beneath Chugoku^6,10^. When the dip angle is greater than 20°, shallow subduction is still obtained relatively independent of convergence rates and plate age (Figs. S7, S8, and S9). The model that shows the best agreement with the PHS slab geometry for the profile shown in Fig. 1B,C,D is reproduced with the convergences rate and age variations of Model 2 (Fig. 3B).

Figure 2 shows that the combination of plate age and reduced initial dip angle of the weak zone generates a low-angle subduction in the first few Myr after the onset of subduction. However, sustaining this low dip angle through time depends on the convergence rate. Hence, Model 1 despite the relatively young plate age of < 20 Myr, the final modeling result is steep subduction. Moreover, our models also indicate that when the plate motion velocity approaches cessation (Fig. 2A), slab pull starts to dominate the system and the final slab geometry is steep subduction (Fig. 3A).

Some studies suggest that the subduction along the Nankai Trough initiated under a very shallow dip angle, due to the warm and buoyancy of the Shikoku Basin^3,22^. Our numerical models indicate that the coupling between the continental plate and PHS plate is strong in the initial phase of subduction (Fig. 2), resulting a shallow slab. In the early stages of subduction, buoyancy forces might have dominated over gravity forces, still, subduction successfully initiated due to a high convergence rate although at a shallow angle^3,22,23,44,45^. The scenario supported by Model 2 finds a close correspondence with the actual slab geometry beneath the Chugoku region (Fig. 3B) and the initiation of subduction at 15 Ma in clear contrast to Model 1 that results in steep subduction (Fig. 3A).

An important role in controlling the dip angle is represented by a combination of local mantle flow conditions, oblique subduction and even the interaction with the PAC slab^46–48^. However, this analysis is of a 3D nature, and represents a numerical limitation of our 2D study. Seismic tomography studies show that the PAC slab is obliquely subducting below Shikoku-Chugoku region and at a depth greater than 500 km^6,10^. Some studies suggest that the PAC slab can disturb locally mantle flow and can influence the dip angle of the PHS slab^48^. However, this effect is dependent on the distance between the PHS slab and the PAC slab^49^. For example, regions near the Sagami Trough are more sensitive to mantle flow caused by double subduction, while in Shikoku region the mantle flow tends to be weak due to the shallow subduction and the distance from the Izo-Bonin-Mariana Trench and Sagami Trough^49,50^. Our models find sensitivity between dip angle and the high-viscosity layer below 500 km representing PAC slab anchored in the transition zone^51–53^ (Figs. S1 and S10), especially for Model 2. Although low-angle subduction is maintained and mantle flow in the mantle wedge is weak, results show that initial dip angles between 20° and 30° generate shallow subduction for Model 2 (Figs. S7, S8 and S9). It is possible that low-angle subduction is influenced by the preferential direction of mantle flow generated by the PAC slab^51^. However, our 2D models are unable to capture this process.

### Magmatism and slab dynamics in the Chugoku and Shikoku regions

In terms of magmatic and plutonic activity associated with the subduction history of PHS plate, there are several controversial aspects related with the nature of MORB-type intrusions located in SW Japan forearc^5,18,22,54^. Actually, felsic and alkaline basalts forming the Setouchi forearc have ages of ~ 14–13 Ma^3,5^ (Fig. 1A), followed by the extinction of volcanism between 12 and 4 Ma and subsequently the formation of monogenetic volcanoes of Quaternary age that can be found in the Chugoku and Shikoku regions^3–5,22^. The origin of current volcanism in Chugoku region has been related with upwelling of hot asthenosphere that interacts with the subducted plate, generating slab melting^4,22^. However, some studies suggest that the shallow dipping slab geometry actually prevents sufficient mantle upwelling to generate volcanic arcs^3^. Other hypotheses suggest melting of the continental crust^3^. One robust line of evidence relies on the formation of a young slab by the opening of the Shikoku Basin which is coeval with subduction initiation along the Nankai Trough at 15 Ma^22^. This type of young slab has a high strong positive buoyancy and resists subduction, but might be facilitated by the preexistence of a fairly low angle weak zone or fault, as confirmed in this study ^3,23,43^. The interaction from 15 Ma to 12 Ma between the young slab and the mantle beneath SW Japan could have produced melting and created the necessary conditions for a magmatic arc composed of felsic-plutonic intrusions^3,5,22^.

The PHS slab located beneath the Chugoku region is characterized by eclogite transformation and slab dehydration a depth of 60 km^3,5^. For a young slab, shallow water release is mainly located below the forearc, where a cold mantle prevents partial melting^30–32^. Some studies have determined that the temperature of the mantle wedge beneath the Quaternary volcanism is in the range of ~ 800°C^48^. This suggests that the present-day volcanism in the Chugoku region, which is distantly located at almost 400 km from the Nankai Trough, may not be caused entirely by slab dehydration^3,48^. Studies show that adakitic magmas, produced by the melting of the lower continental crust in the presence of plagioclase and amphibole, may be contributing to the volcanic activity in the region^3^. Other studies show the presence of a large low-velocity anomaly located beneath the Chugoku district, which may indicate an upwelling mantle anomaly, which affects the PHS slab edge, ultimately producing slab melting^4,22,26^.

Although the origin of Quaternary-age volcanism is controversial beneath Chugoku, our numerical models can provide some useful insights regarding the evolution and nature of the volcanic arc in the study region^6,31^. Figure 2 shows prediction after 15 Myr of evolution for two numerical models, reproduced under two distinctive dynamic scenarios. Subduction models that predict a steep slab angle (Model 1, Fig. 3A) tend to have high temperatures in the mantle wedge. These conditions are more favorable for the formation of a volcanic arc close to the trough, which is observed in several regions such as Kyushu in southwestern Japan, Central America, Aleutian Islands, as well as northeastern Japan^30–32^.

On the other hand, models obtained based on high convergence rates between 15 Ma and 3 Ma (Fig. 3B), show a predisposition to shallow subduction with a slab geometry similar to that observed in the Shikoku and Chugoku districts^6,10^ (Fig. 3B).

Although, our models do not incorporate partial melting, we can predict the position of a volcanic arc based on the slab geometry. The subduction of a young and buoyant slab in conjunction with a low subduction angle prevents the formation of volcanic arcs near the trough^36,55^. This is consistent with global observations where the volcanism migrates towards the interior of the continental plate when the dip angle decreases, such is the case of Trans-Mexican-Volcanic arc (TMVA) in Central Mexico^36–38^.

**Figure 4:**
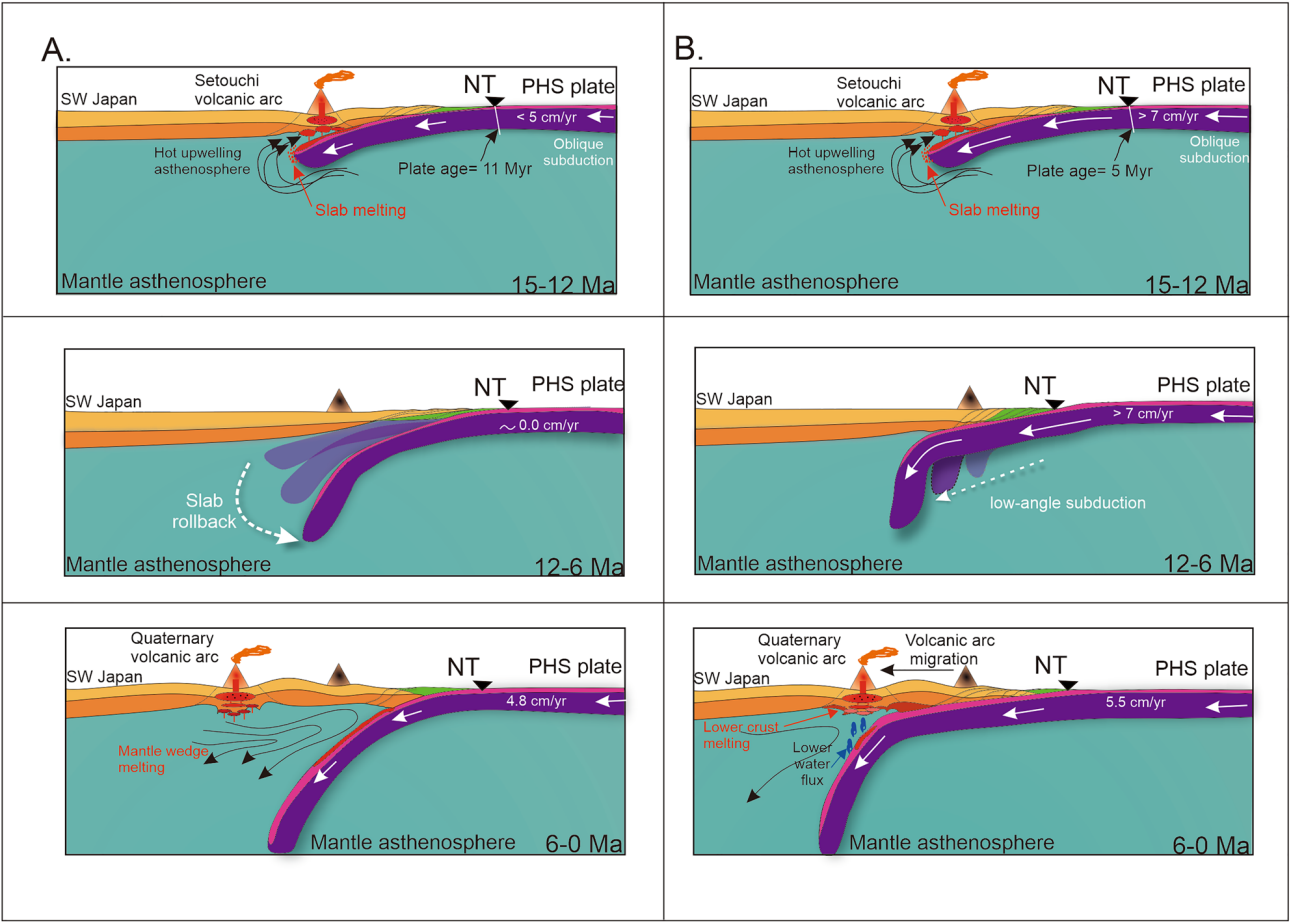
Graphical representation of evolutionary history of the PHS slab based on the two models of tectonic evolution (Fig. 3A,B). (**A**) Subduction history under the hypothesis of slow subduction motion between ~ 11 Ma and 7 Ma. (**B**) Subduction history for a high convergence rate between ~ 15 Ma and 3 Ma. This figure is created using Corel Draw 2022.

The schematic view for subduction evolution for Models 1 and 2 that incorporates a possible location of the volcanic arc can be useful to better interpret modeling results. Figure 4 shows a synthesis of the evolution of the PHS slab based on the two settings presented in this work (Fig. 2). According to our numerical simulations, slab evolution is rather different. Subduction initiation induces a mantle flow which mobilizes subslab hot asthenosphere material to flow around the tip of the young PHS slab. The volcanic arcs can occur as a consequence of this peculiar slab mantle interaction, and is probably enhanced by an upwelling mantle that occurs during Japan Sea opening before 15 Ma^4,22^. However, significant difference between subduction styles is observed after 11 Ma (Fig. 4A,B). Model 1 suggests that volcanism disappears between ~ 11 Ma and 7 Ma due to the cessation of subduction^4,22^, and slab starts bending into the mantle owing to the dominance of gravity slab pull forces. On the other hand, Model 2 shows (Fig. 4B) the evolution of the PHS slab during 15 Ma to the present a which is characterized by long period of high convergence rate between 15 Ma and 3 Ma^3^ (Fig. 2B). The prolonged high convergence rate and the positive buoyancy of the slab produces initially a low-angle subduction that is maintained during this period. In this case, the cessation of volcanism between 12 Ma and 4 Ma is directly related to the low angle and the impossibility of generating sufficient fluids that can reach the distant mantle wedge from the trough axis ^55^. Although it is not clear how volcanism is reactivated after 4 Ma, our hypothesis based on Model 2 is that low-angle subduction displaces volcanism towards the Japan Sea coast, which is consistent with the position of the Sanbe volcano^3,55^ (Figs. 1, 3B, 4B). This is a process similar to the evolution of the Trans-Mexican Volcanic Belt that changed its position as a result of slab flattening at ~ 13 Ma^36–38^.

### Numerical model limitations

Our 2D numerical models do not consider double subduction together with the PAC plate, phase transitions at depths of 410 km and 660 km, and 3D mantle flow effects possibly associated with oblique subduction. However, the interaction of the PHS slab with the PAC slab is highly controversial. 3D numerical studies have shown that the oblique subduction and the flow patterns generated by the double subduction affect the geometry of the slab^46–48^. Although our plate motion velocity calculations consider oblique change of direction in the subduction of the PHS plate with respect to the Amurian plate (AM) and its separation with the Eurasian plate^13,16^, a generalized 3D numerical study is needed for Nankai Trough, which includes the PAC plate subduction dynamics. The PHS plate motion changes and oblique subduction of the PHS plate determines whether the tectonic scenario proposed by Tatsumi et al. (2020)^3^ continues to generate slab geometries consistent with the current slab geometry beneath the Shikoku and Chugoku regions.

Our Model 2 can explain several key observations, as the disappearance of volcanism between 12 Ma and 3 Ma, the onset of subduction along the Nankai Trough, and the shallow subduction beneath the Shikoku and Chugoku regions^3,6,7,55^. On the other hand, our numerical results report high rates of advance slab, which is somehow inconsistent with other studies in the region^55^. However, there are several studies that report both, trench retreat and advance processes for the last 15 Myr^48,53,56^. These processes may affect the dip angle of the slab^57^. Our numerical models are consistent with the kinematics proposed by the high convergence hypothesis.

The behavior of the state of stress due to the onset of oblique subduction along SW Japan cannot be analyzed with our 2D numerical models, this analysis is of 3D nature. Only the compressional stress direction perpendicular to the trench can be reported by our numerical models (Fig. S11), which are consistent with high compression reported in SW Japan^58,59^. This is mainly due to high convergence of the PHS plate^58,59^. However, there is a relatively short period of 3 Myr (between 15 Ma and 12 Ma) when the Setouchi volcanic arc is characterized by normal extension as a consequence of its formation^4,5,58,59^. After 12 Ma, when volcanism ceases, the Setouchi volcanic arc reports strong compression^58,59^. This is recorded, for example, in the Kumano pluton on the Kii peninsula after its formation^4,59^. Recently, the stress state is affected again in the Quaternary, due to the change of direction of the PHS plate movement and the reactivation of volcanism^25,58^. Due to the 2D nature of our models, they cannot capture the change in stress direction caused by the PHS plate changing direction at 3 Ma^3^. Another limitation is related with the Setouchi volcanic arc formation and pluton emplacement that occurs between 14 Ma and 13 Ma^4,5^, and therefore, our models are not consistent with this extensional period. A future 3D study that incorporates slab melting is proposed to determine if the hypothesis proposed by Tatsumi et al. (2020)^3^ remains consistent with paleo-stress between 14 Ma and 13 Ma and for after the Quaternary.

## Conclusions

Using thermomechanical visco-elasto-plastic codes that incorporating spontaneous subduction, we investigate how subduction history affects the style of subduction along the profile passing through the Shikoku and Chugoku districts in southwest Japan, which is almost perpendicular to Nankai Trough. We analyze the subduction evolution of the Philippine Sea slab since 15 Ma incorporating different trough-normal temporal change in convergence rates and age proposed in previous studies. Since our simulations include subduction initiation, we include as a free parameter the initial dip angle of a mechanically weak zone that allows subduction to initiate and subsequently freely develop. We compare our predictions with the observed current PHS slab geometry and, at a certain extent, with the distribution of volcanism. Numerical models show that the combination of convergence rate and age of the PHS plate can significantly affects the slab dynamics. The numerical simulation that best approximates the present-day slab geometry proposed by Hirose et al. (2008)^10^ below the Shikoku and Chugoku regions is obtained using the Tatsumi et al. (2020) tectonic model^3^ (Model 2). In this case for the subduction initiation, we use a simulation with a low initial dip angle of the weak zone of 20°. Such a fairly low angle of the initial thrust subduction fault might be previously formed due to the rotation of the PHS plate during the Japan Sea opening^1,2,15–17^ before 15 Ma. On the other hand, the model that incorporates the evolutionary scenario of Kimura et al. (2014)^4^ produce a slab geometry with a greater dip angle than what is currently observed. Since the development of volcanism is strongly related to the style of subduction (i.e., slab dip), our models indicate that the high convergence rate and buoyancy of the young slab, produces subduction with an extremely low dip angle, and therefore, the extinction of volcanism between 12 Ma and 3 Ma may be associated with a low angle of subduction and not necessarily with the cessation of subduction.

## Methods

For our numerical modeling, we used the freely available visco-elasto-plastic subduction Matlab code ^33,34^ and modified it to incorporate time-dependent boundary conditions depending on the variations of convergence rates. We also incorporate variable PHS plate ages at the Nankai trough and variable initial dip angle of a weak zone that facilitates subduction initiation. The mass, momentum and energy equations are solved in a 2D Cartesian domain under the adaptive mesh refinement methodology using the finite difference method and particle-in-cell technique^33,34^. The computational domain has a total size of 2500 × 600 km with 301 x-nodal points and 151 y-nodal points and the domain of interest is shown in Fig. S1. In addition, our models incorporate a high discretization between [850–1150 km] in horizontal direction and [0–150 km] in vertical direction. The domain of interest is highly discretized and is divided into 150 nodal points in the x-direction and 102 nodal points in the y-direction (Fig. S1A). The high discretization helps to better represent the high strain rates that are concentrated along the plate interface. The oceanic plate is composed of sediments, basaltic and gabbroic layers, with their respective radiogenic heat release values and plasticity conditions shown in Tables S3, S4, S5 and S6. The model incorporates a sticky air layer in the uppermost part that acts as an internal free boundary condition avoiding deformation resistance and oscillations in the solution that can led to numerical instabilities^33,34^ (Fig. S1B). The changes of thickness for the PHS slab along the profile passing through the Shikoku and Chugoku regions is calculated with the following formulation^60^

1 $$2.3\sqrt{\mathrm{\kappa t}}$$

where κ is thermal diffusivity (10^–6^ m^2^ s^−1^) and t is the plate age (seconds) at the trough^60^. Between the continental and the oceanic plates, a mechanically weak zone is incorporated that allows spontaneous plate bending^33^. In this region the initial slab depth is 80 km (Figs. S1B and C). This zone deforms as a subduction fault with brittle and ductile behavior and imposes rheological conditions of wet olivine with a low plasticity^33,34^ $(\mathrm{sin}\left(\varphi \right)=0$). The inclination of the zone of weakness is defined by the initial dip angle and in our models, we use angles between 15° and 30°. The continental plate temperature distribution varies with depth as a linear geothermal gradient, whereas for the oceanic plate temperature the temperature gradient varies with respect to the age and changes with time. In our numerical models the continental plate has a constant thickness of 35 km^7,8,35^ (Fig. S1B). The right boundary has an imposed convergence rate variation tailored to each of the two tectonic scenarios investigated in this work (Fig. 2A,B). The other boundaries have a free-slip type condition that ensures the flow velocity normal to the boundary is zero and that the other components do not change across the boundary^33,61^. The slab is subjected to two types of mechanisms, the horizontal velocity imposed on the right side of the boundary (Fig. S1) and by slab pull due to density and temperature changes between the subducted oceanic lithosphere and the mantle^33^. In the latter case, the slab experiences resistance forces represented by flexure, buoyancy and viscosity as the slab interacts with the mantle^33,61^

### Supplementary Information

Supplementary Information 1.

Supplementary Video 1.

Supplementary Video 2.

## Data Availability

The datasets used and/or analyzed during the current study are available from the corresponding author on reasonable request. Slab geometry is obtained from https://www.mri-jma.go.jp/Dep/sei/fhirose/plate/en.PHS.html. Seismic tomography is available from https://www.hinet.bosai.go.jp/topics/sokudo_kozo/alljpn_download.php. The LFES and hypocenter information is available on https://www.data.jma.go.jp/svd/eqev/data/bulletin/hypo_e.html. Tectonic Tremors information is available from http://www-solid.eps.s.u-tokyo.ac.jp/~sloweq/?page=map.
