# Supplementary material for: Numerical modeling of subduction and evaluation of Philippine Sea Plate tectonic history along the Nankai Trough
Source: Sci Rep. 2023 Oct 25;13:18313. doi: 10.1038/s41598-023-45370-2 (PMC10600142; doi:10.1038/s41598-023-45370-2)
Supplement: Supplementary file 1 — Supplementary Information 1. [file 41598_2023_45370_MOESM1_ESM.docx]

Supplementary Information for

**Numerical modeling of subduction and evaluation of Philippine Sea plate tectonic history along the Nankai Trough**

E. J. Moreno^1^, V.C. Manea^1,2*^, M. Manea^1,2^, S. Yoshioka^1,3^, N. Suenaga^4^ and A. Bayona^2^

^1^ Research Center for Urban Safety and Security, Kobe University, Kobe 657-8501, Japan

^2^ Computational Geodynamics Laboratory, Centro de Geociencias, Universidad Nacional Autónoma de México, Campus Juriquilla, Querétaro, 76230, México

^3^ Department of Planetology, Graduate School of Science, Kobe University, Kobe 657-8501, Japan

^4^ Research Center for Earthquake Hazards, Disaster Prevention Research Institute, Kyoto University, Kyoto 611-0011, Japan

* Corresponding author: [vlad@geociencias.unam.mx](mailto:marina@geociencias.unam.mx)

**Contents of this file**

1. Supplementary model settings and parameters
2. Model setup and Boundary conditions.
3. Supplementary model simulations
4. Supplementary animations
5. Supplementary References
6. **Supplementary model settings and parameters**
   1. **Introduction**

In our study we investigate the Philippine Sea (PHS) plate for the last 15 Myr using two tectonic hypotheses based on the variation of plate motion velocity and age at the Nankai Trough. The first scenario is based on the migration of the Nankai Trough, Japan Trench and Izu-Bonin Mariana Trench triple junction. In this case, the PHS plate initiated its subduction with an age at the trough of ~11 Ma and evolved afterwards with a near to zero convergence rate between ~11 Ma and 7 Ma^1^. The second hypothesis is based on the nature and distribution of volcanism on the Chugoku region with respect to the PHS slab dynamics. In this case, the PHS plate initiated the subduction with a plate age of ~5 Myr and convergence rate normal to the Nankai Trough of ~7.33 cm/yr. The high convergence rate is maintained between 15 Ma and 3 Ma^2^. Subduction evolution is integrated over a period of 15 Myr, and the final results are compared with observed current slab geometry for a profile passing through the Shikoku and Chugoku regions^3,4,5^ (Fig. 1).

**1.1.1 Numerical modeling setting**

To investigate how subduction parameters (plate motion velocity and age) affect the dynamics of the PHS slab in the last 15 Myr, we employ high-resolution 2D visco-elasto-plastic numerical models of spontaneous subduction. Our numerical models use the mass, momentum and energy conservation equations with a mixed Lagrangian-Eulerian numerical scheme based on conservative finite-differences and marker-in-cell techniques^6,7^.

The conservation of mass is approximated by time-dependent continuity equation:

$$\frac{Dln\rho}{Dt}+\frac{\partial v_{x}}{\partial x}+\frac{\partial v_{y}}{\partial y} (1)=0 (1)$$

where $\frac{D}{Dt}$ is the substantive time derivate and$\rho(T,P,C)$ is depending on the temperature, pressure and composition.

The second equation in our models involves a non-Newtonian rheology which will depend on elastic, viscous and plastic deformations.

$\frac{\partial\sigma_{xx}}{\partial x}+\frac{\partial\sigma_{xy}}{\partial y}-\frac{\partial P}{\partial x}+\rho g_{x}=\rho\frac{Dv_{x}}{Dt}$ (2)

$\frac{\partial\sigma_{yy}}{\partial y}+\frac{\partial\sigma_{yx}}{\partial x}-\frac{\partial P}{\partial y}+\rho g_{y}=\rho\frac{Dv_{y}}{Dt}$ (2.1)

The density is expressed as:

$\rho=\rho_{r}\frac{1+\beta(P-P_{r})}{1+\alpha(T-T_{r})}$ (2.2)

where $\rho_{r}$ is the density for a material at reference pressure $P_{r}$ $\left( {10}^{5} \mathrm{Pa} \right)$ and temperature $T_{r} (298.15 K)$, $\alpha$ and $\beta$ are thermal expansion and compressibility coefficients, respectively.

The energy conservation, when the creeping flow, both thermal and chemical buoyancy are incorporated, is expressed as^6,7,8,9^:

$\rho C_{p}\left( \frac{DT}{Dt} \right)=-\frac{{\partial q}_{x}}{\partial x}-\frac{{\partial q}_{y}}{\partial y}+H_{r}$+ $H_{a}$+ $H_{s}$ (3)

$q_{x}=-T,P,c)$ $\frac{\partial T}{\partial x}$, $q_{y}=-T,P,c)$ $\frac{\partial T}{\partial y}$ (3.1)

$H_{a}=T\alpha\left( v_{x}\frac{\partial P}{\partial x}+v_{y}\frac{\partial P}{\partial y} \right)$ (3.2)

$H_{s}=\sigma_{xx}\left( \dot{\varepsilon}_{xx}-\dot{\varepsilon}_{xx\left( elastic \right)} \right)+$ $\sigma_{yy}\left( \dot{\varepsilon}_{yy}-\dot{\varepsilon}_{yy\left( elastic \right)} \right)+2\left( \dot{\varepsilon}_{xy}-\dot{\varepsilon}_{xy\left( elastic \right)} \right)$ (3.3)

where $H_{r}$, $H_{a}$ and $H_{s}$ represent radioactive, adiabatic (changes of pressure) and shear heating (dissipation of the mechanical energy during irreversible no-elastic deformations), respectively. $T,P,c)$ is the thermal conductivity and depends on pressure, temperature and rock composition, $\alpha$ is the thermal expansion coefficient, and *C* is rock composition^6,7,8,9^.

**1.1.2 Visco-elasto-plastic rheology**

Considering a realistic visco-elasto-plastic rheology is a key to understand the slab deformation from subduction initiation and subsequent processes of slab bending^6,7,9^. Based on the assumption that viscous, elastic, and plastic deformations develop under the same applied deviatoric stress, and the plastic formulation is the same for both dilatant and non-dilatant materials, the bulk deviatoric strain rate can be represented as a sum of viscous, elastic, and plastic strain rate^9^:

${\dot{\varepsilon}'}_{ij}={\dot{\varepsilon}'}_{ij(viscous)}+{\dot{\varepsilon}'}_{ij(elastic)}+{\dot{\varepsilon}'}_{ij(plastic)}$ (4)

where

$\dot{\varepsilon}_{ij(elastic)}=\frac{1}{2\mu}\frac{D\dot{\sigma}_{ij}}{Dt}$ (4.1)

$\dot{\varepsilon}_{ij(viscouss)}=\frac{1}{2\eta}\dot{\sigma}_{ij}$ (4.2)

$\dot{\varepsilon}_{ij\left( plastic \right)}=0 \mathrm{fo}r \sigma_{II}<\sigma_{yield}$ (4.3)

$\dot{\varepsilon}_{ij\left( plastic \right)}= \chi\frac{\dot{\sigma}_{ij}}{2\sigma_{II}} \mathrm{for} \sigma_{II}=\sigma_{yield}$ (4.4)

$\sigma_{II}=\sqrt{\frac{{\dot{\sigma}_{ij}}^{2}}{2}}$ and $\sigma_{yield}=c+\sin\varphi*P.$ (4.5)

where $\dot{\varepsilon}_{ij}$ is the strain rate, *η* the viscosity, and *μ* the shear modulus, $\frac{D\dot{\sigma}_{ij}}{Dt}$ is the objective co-rotational time derivative of the deviatoric stress component $\dot{\sigma}_{ij}, \sigma_{II}$is the second invariant of the deviatoric stress tensor, *χ* is the plastic multiplier (unknown a priori), which satisfies the plastic yielding condition $\sigma_{II}=\sigma_{yield}$. The plastic multiplier is a variable scaling coefficient, which connects components of the plastic strain with the deviatoric stress components when the yielding condition is reached, *c* is the rock cohesion,$\varphi$ is an angle of internal friction, *P* is pressure, and $\dot{\varepsilon}_{II\left( plastic \right)}$ is the second invariant of the deviatoric plastic strain rate tensor^6^. $\sigma_{yield}$ and *χ* are calculated at each time step and allow the decoupling of the continental and oceanic plates^6,9,10^.

In our simulations the different mechanisms such as diffusion and dislocation creep are represented by the sum of each component and in this case the viscosity formulation is:

$\eta=\frac{\eta_{df}\eta_{ds}}{\eta_{df}{+\eta}_{ds}}$ (5)

where

$\eta_{df,ds}=\left( \frac{d^{p}}{AC_{OH}^{r}} \right)^{\frac{1}{n}}\dot{\varepsilon}_{E}^{\frac{1-n}{n}}exp\left[ \frac{E^{*}+PV^{*}}{nRT} \right]$ (5.1)

where *d* is the gran size, $p$ is grain size exponent, $r$ is $C_{OH}$ exponent, *A* is the pre-exponential factor, *C_OH_* is *OH* concentration, *n* is the stress exponent (non-Newtonian rheology) and $\dot{\varepsilon}_{E}=\frac{1}{2}\dot{\varepsilon}_{ij}\dot{\varepsilon}_{ij}^{1/2}$ is the effective strain rate ^6,7,8,11^.

1. **Supplementary Figure S1. Model setup and Boundary conditions**

Supplementary Figure S1 shows the model configuration of our numerical simulations. The model includes a hot segment of continental lithosphere near the trough representing the SW Japan continental crust. Also, to facilitate subduction initiation, we introduce a weak zone (with dip angles of 15°-30°) between the oceanic and continental plates^12^. The slab thickness changes with respect to time and are defined with respect to the age and plate motion velocity distributions shown in Figs. 2A and 2B. The subduction initiation for Models 1 and 2 is influenced by the angle imposed on the weak zone and by the plate motion velocities and ages at 15 Ma shown in Tables S1 and S2. The structure of the slab involves evolution of sediment, basaltic and gabbroic layers, and the continental plate is represented by the upper and lower crust. The rheological properties are defined according to the flow laws discussed in Supplementary Section 1.1.2, and the rheological characteristics are presented in Tables S3, S4, S5 and S6. Our numerical models do not incorporate the kinematic evolution of Pacific (PAC) plate, instead we impose a low temperature with high viscosity layer (viscosity of $1\times{10}^{23}$ Pa s and temperature of ~900°C) below 500 km depth ^3,4,5^  and we assume that the PAC slab has remained anchored in this area during the entire simulation period of 15 Myr^13,14^.

| 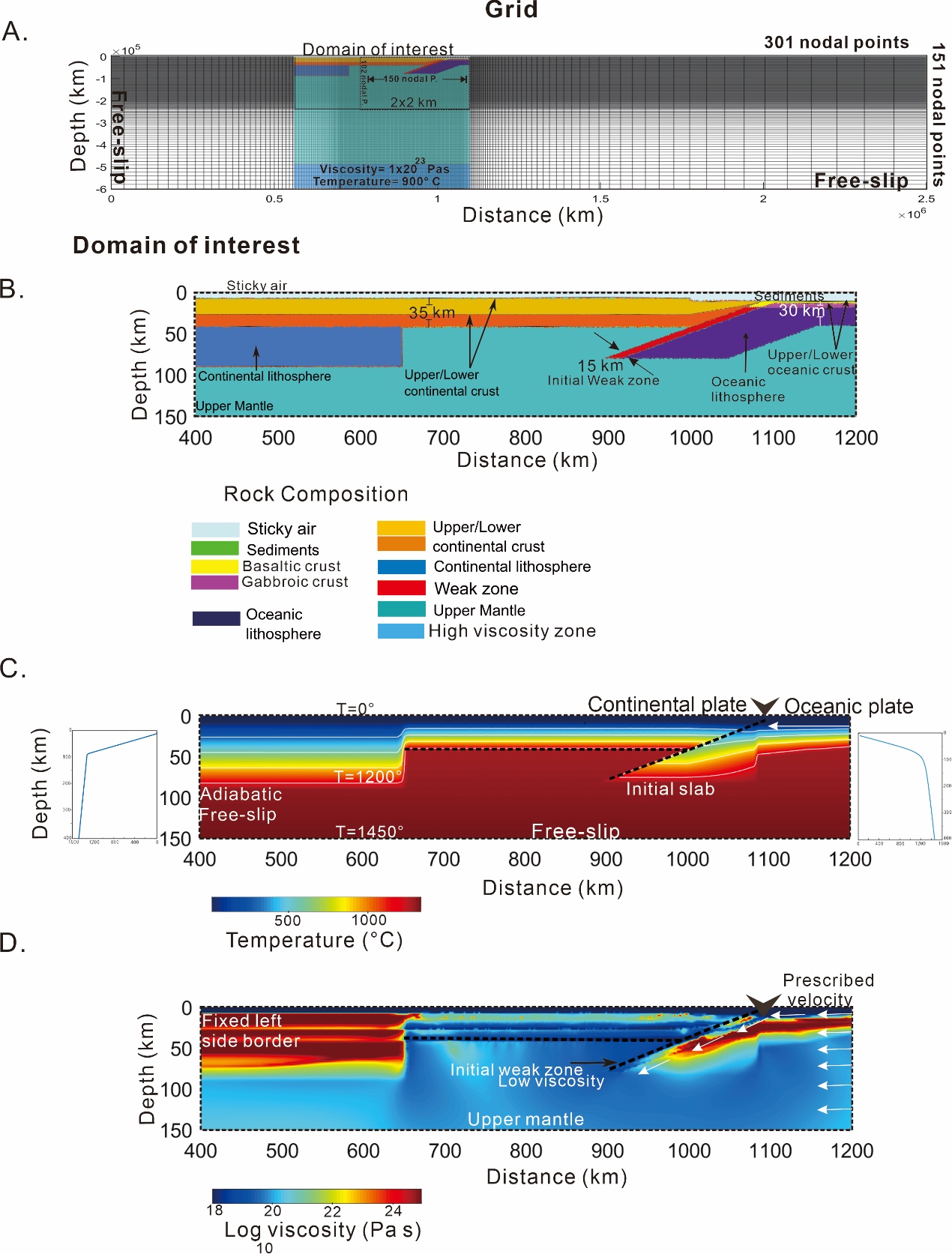 |
| --- |
| **Fig S1.** Model setup and boundary conditions: A) Mesh discretization, B) Rock composition, C) Temperature and D) Viscosity. The temperature structure for the slab uses a semi-infinite half-space cooling and it is age dependent^6,12^. The continental crust is constructed with a linear temperature gradient, incorporates radiogenic heat production^6,12^. The right side of the 2D model domain evolves according to the time variable plate motion velocity imposed. On the left side of the computational domain, the model incorporates an adiabatic gradient profile with a free-slip boundary which ensures that the stress normal to the surface is zero. At the top an isotherm is imposed with T=0°C and in the lower part of the model, the isotherm takes a value of T=1450°C with free-slip boundary condition^6^. We also include an adiabatic gradient of 0.5 °/km below the lithosphere in the mantle^6^. The right boundary changes position at each time step in response to convergence rate of the PHS plate. The model incorporates a sticky air layer in the upper part. Sticky air layer, characterized by low density and viscosity (water density: 1000 kg/m^3^) is implemented in the upper part of the model^6^. Sticky air behaves as a free internal boundary to ensure deformations at very low stress behaving as an internal free boundary condition and avoiding deformation resistance and oscillations in the solution that can produce numerical instabilities^6^. In order to satisfy the conservation of mass equation and ensure correct convergence of the system of equations, the model domain becomes thicker proportionally to the displacement and shortening of the right boundary^6^. The minimum viscosity in the lower part of the model is ${1\times10}^{18}$ Pa s and the maximum viscosity in the upper part of the model is ${1\times10}^{25}$ Pa s. The initial weak zone is defined with respect to the rheology of the olivine wet with a brittle-plastic strength of 1 MPa^6^. |

- 1. **Velocity and age history for the PHS slab**

The velocity and age for Model 1 and Model 2 are projected to the profile across the Chugoku region using the methodology described by Yoshioka et al. (2013) ^15,16^. The calculation of velocities and ages assumes the initiation of subduction at 15 Ma^17,18,19^ and the oblique change of direction in the subduction of the PHS plate with respect to the Amurian (AM) plate and its separation from the Eurasian (EU) plate at 3 Ma^21,21,22^. The age is calculated in the following equation:

$t_{s}=\left( t_{calc}+\frac{\left| X-\int_{0}^{t} v_{s}(t)dt \right|}{v_{r}} \right)\times{10}^{-6}$ (6)

where $t_{calc}$ (yr) is the elapsed time since the initiation of the PHS plate subduction, $X$ is the distance between the fossil ridge axis at 15 Ma and the starting point of the profile that passes through the Shikoku and Chugoku regions, $v_{s}(t)$ (cm/yr) is the temporal migration rate of the PHS plate from 15 Ma to the present and where the direction is projected perpendicular to the profile, and $v_{r}$ is the expansion rate of the Shikoku Basin, which was determined to be 3.12 cm/yr^15,16^.

Our numerical models aim to analyze subduction dynamics incorporating plate age and plate motion velocity, two hypotheses^1,2^ (Models 1 and 2) that propose different conditions for the evolution of the PHS plate along the Nankai Trough. Time variations of convergence rates and plate ages are shown in Figs. 2A and 2B. Velocities and ages at 15 Ma and present are shown in Tables S1 and S2 for Models 1 and 2.

**Table S1: Convergence rate and age values for Model 1 at 15 Ma and at present.**

| **Time (Ma)** | **Convergence rate (cm/yr)** | **Age (Myr)** |
| --- | --- | --- |
| t = 15 | 4.24 | 11 |
| t = 0 | 4.88 | 17.36 |

**Table S2: Convergence rate and age values for Model 2 at 15 Ma and at present.**

| **Time** | **Convergence rate (cm/yr)** | **Age (Myr)** |
| --- | --- | --- |
| t = 15 Ma | 7.33 | 5.1 |
| t = 0 Ma | 5.57 | 17.36 |

**Table S3: Thickness of the layers that conform the PHS plate and SW Japan.**

| **SW Japan** | **Thickness (km)** | **PHS plate** | **Thickness (km)** |
| --- | --- | --- | --- |
| Upper continental crust | 20 | Sediments | 1 |
| Lower continental crust | 15 | Basaltic crust | 2 |
|  | | Gabbroic crust | 5 |
|  |  | Lithosphere | 22 |

**Table S4**. Material properties used in numerical experiments^6,8,12^.

| Material | Density  (kg/m^3^) | Thermal conductivity  (W/m K) | Rheology  Ea (kJ/mol), A_D_ (MPa^-n^s^-1^), c (MPa) | Heat capacity  (J/kg K) |
| --- | --- | --- | --- | --- |
| Sticky air layer | 1000 (solid) | 300 | Constant viscosity: 10^18^ Pa s | 3000 |
| Sedimentary rocks | 2700 (solid) | 0.64+807/(T-77); where T is temperature in (K). | Power flow law, n=2.3, Ea=154, A_D_=3.2x10^-4^, | 1000 |
| Basaltic crust | 3200 | 1.18+474/(T-77); | Power flow law, n=2.3, Ea=154, A_D_=3.2 x10^-4^, | 1000 |
| Gabbroic crust | 3200 | 1.18+474/(T-77); | Power flow law, n=3.2, Ea=238, A_D_=3.3 x10^-4^, | 1000 |
| Lithosphere | 3300 | 0.73+1293/(T-77); | Power flow law, n=3.5, Ea=532, A_D_=2.5 x10^-4^, | 1000 |
| Weak zone | 3300 | 0.73+1293/(T-77); | Power flow law, n=4.0, Ea=471, A_D_=2.0 x10^-4^, | 1000 |
| Upper continental crust (quartzite wet) | 2800 | 0.64+807/(T-77); | Power flow law, n=2.3, Ea=154, A_D_=3.2 x10^-4^, | 1000 |
| Lower continental crust (plagioclase An_75_) | 2900 | 0.73+474/(T-77); | Power flow law, n=3.2, Ea=238, A_D_=3.3 x10^-4^, | 1000 |

**Table S5. Material properties used in numerical experiments**^6,8,12^**.**

| Material | Shear modulus  (Pa) | Thermal expansion (1/K) | Compressibility  (1/Pa) | Radioactive heating (mW/m^3^) |
| --- | --- | --- | --- | --- |
| Sticky air layer | 1x10^20^ | 3x10^-5^ | 1x10^-11^ | 0 |
| Sedimentary rocks | 1x10^10^ | 3x10^-5^ | 1x10^-11^ | 2 |
| Basaltic crust | 2.5x10^10^ | 3.5x10^-5^ | 1x10^-11^ | 2.5 |
| Gabbroic crust | 2.5x10^10^ | 3.5x10^-5^ | 1x10^-11^ | 2.5 |
| Lithosphere | 6.7x10^10^ | 3.5x10^-5^ | 1x10^-11^ | 0.28 |
| Weak zone | 6.7x10^10^ | 3.5x10^-5^ | 1x10^-11^ | 0.28 |
| Upper continental crust (quartzite wet). | 1x10^10^ | 3x10^-5^ | 1x10^-11^ | 10 |
| Lower continental crust (plagioclase An_75_). | 2.5x10^10^ | 3.5x10^-5^ | 1x10^-11^ | 5 |

**Table S6. Rheological parameters used in numerical experiments**^6^.

| **Layer** | **Plasticity:** ${\boldsymbol{\sigma}\text{ }}_{\boldsymbol{yield}}\mathbf{=}\boldsymbol{C}\mathbf{+}\sin\left( \right)\mathbf{*}\boldsymbol{P}$**; C (Pa)** |
| --- | --- |
| Sediments | C=10^6^, $\sin\left( \right)$=0.03 |
| Basaltic crust | C=10^6^, $\sin\left( \right)$=0.03 |
| Weak zone | C=10^6^, $\sin\left( \right)$=0.03 |
| Gabbroic crust | C=10^6^, $\sin\left( \right)$=0.03 |
| Lithosphere | C=10^6^, $\sin\left( \right)$=0.6 |
| Upper continental crust | C=10^6^, $\sin\left( \right)$=0.2 |
| Lowe continental crust | C=10^6^, $\sin\left( \right)$=0.2 |

**3. Supplementary model simulations**

We test the sensitivity of our numerical simmulations against the intial dip angle of the weak zone (Fig. S1). In this section we present time evolution for numerical models obtained with initial dip angles for the weak zone between 15°-30°. The models reproduced are performed using plate motion velocities and ages shown in Figs. 2A and 2B. Our models predict that for initial dip angles between 15°-30° steep subduction is obtained for Model 1. On the other hand, for initial dip angles less than or equal to 30°, subduction varies between flat (15°) and shallow subduction (20°-30°) for Model 2. We compare our predictions in term of the observed current slab geometry for a profile passing through the Shikoku and Chugoku regions in the Nankai subduction zone^3,4,5^ (Figs. 1B, 1C and 1D). The results that best approximate the geometry of the slab below Shikoku and Chugoku regions plotted in black in all final steps are those that incorporate as input parameters in Fig. 2B, corresponding to a high convergence rate at the Nankai Trough between ~7-3 Myr.

**Table S7: Initial dip angle and final subduction style for Model 1 and Model 2**

| **Model 1**  **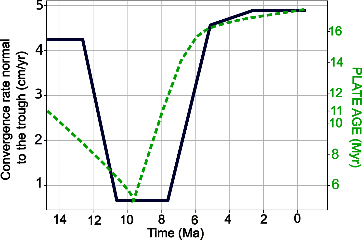** | **Initial Dip Angle** | **Present-day slab geometry** |
| --- | --- | --- |
|  | 15° | Steep slab |
|  | 20° | Steep slab |
|  | 25° | Steep slab |
|  | 30° | Steep slab |
| **Model 2**  **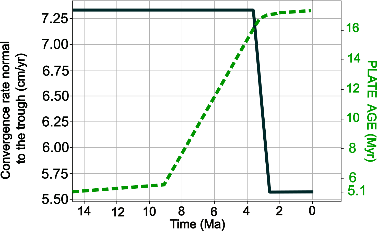** | 15° | Flat slab |
|  | 20° | Shallow slab |
|  | 25° | Shallow slab |
|  | 30° | Shallow slab |

**3.1 Simulations with plate motion velocity and age according to Model 1.**

Supplementary Figs. S2, S3, S4, and S5 show time evolution with temperature, viscosity and composition for a slab evolving for 15 Myr with plate motion velocity and age distribution based on Fig. 2A and initial dip angles for the weak zone between 15°-30° (Table S7). The thickness of the slab changes in time according to age shown in Fig. 2A. Our numerical models that incorporate a slow convergence rate (close to cessation) between ~11 Ma and 7 Ma^1^ and plate age variation at the trough according to Fig. 2A, predict steep slabs (Figs. S2, S3, S4, and S5) that are not consistent with the observed current slab geometry below the Shikoku and Chugoku regions^3^.


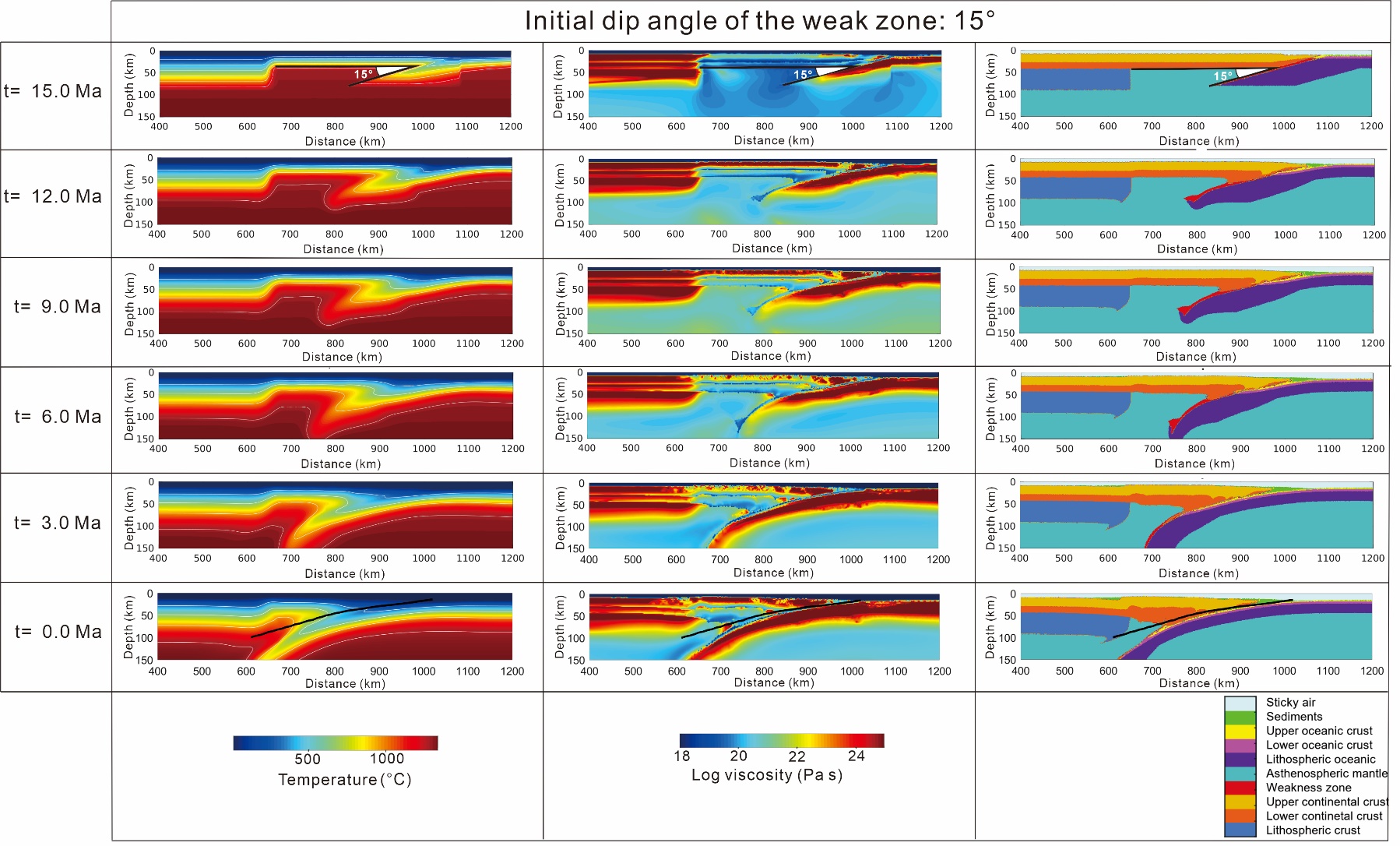


**Figure S2.** Time evolution showing results with a plate motion velocity and age imposed on the PHS plate based on convergence rate and age changes shown in Fig. 2A and initial dip of the weak zone of 15°. In this hypothesis, the PHS slab moves with rates close to zero between ~11- 7 Ma and initiates subduction with an approximate plate age of ~11 Ma^1^. Our models obtain steep subduction by imposing an initial dip angle of 15° for the weak zone. The black curve represents the slab geometry plotted over the final time step, and these values are obtained from https://www.mri-jma.go.jp/Dep/sei/fhirose/plate/en.Tools.html^3,4,5^.

**
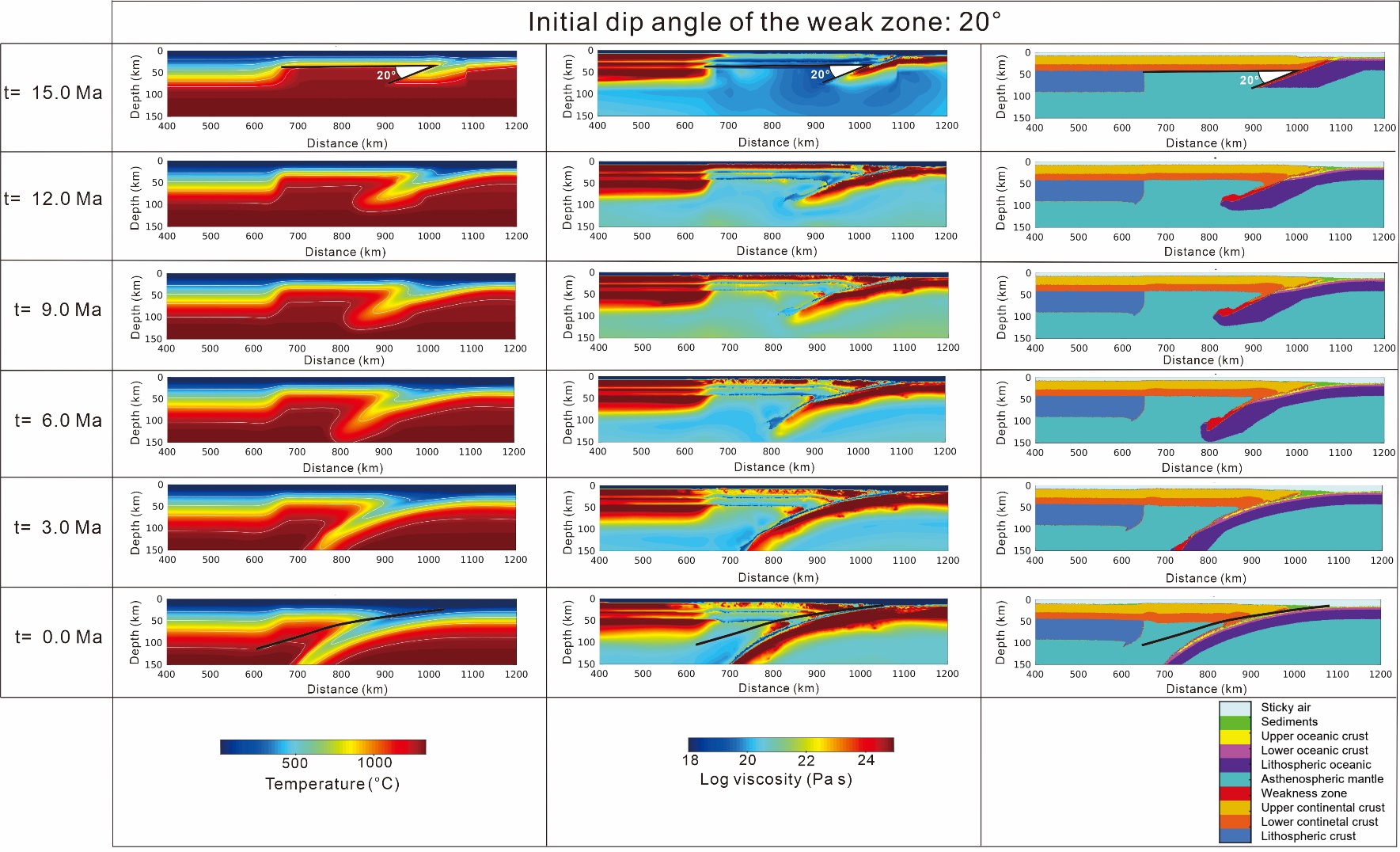
**

**Figure S3.** Time evolution showing results with a plate motion velocity and age imposed on the PHS plate based on convergence rate and age changes shown in Fig. 2A and initial dip of the weak zone of 20°. In this hypothesis, the PHS slab moves with convergence rates close to zero between ~11- 7 Ma and initiates subduction with an approximate plate age of ~11 Ma^1^. Our models obtain steep subduction by imposing an initial dip angle of 20° for the weak zone. The black curve represents the slab geometry plotted over the final time step, and these values are obtained from https://www.mri-jma.go.jp/Dep/sei/fhirose/plate/en.Tools.html^3,4,5^.

**
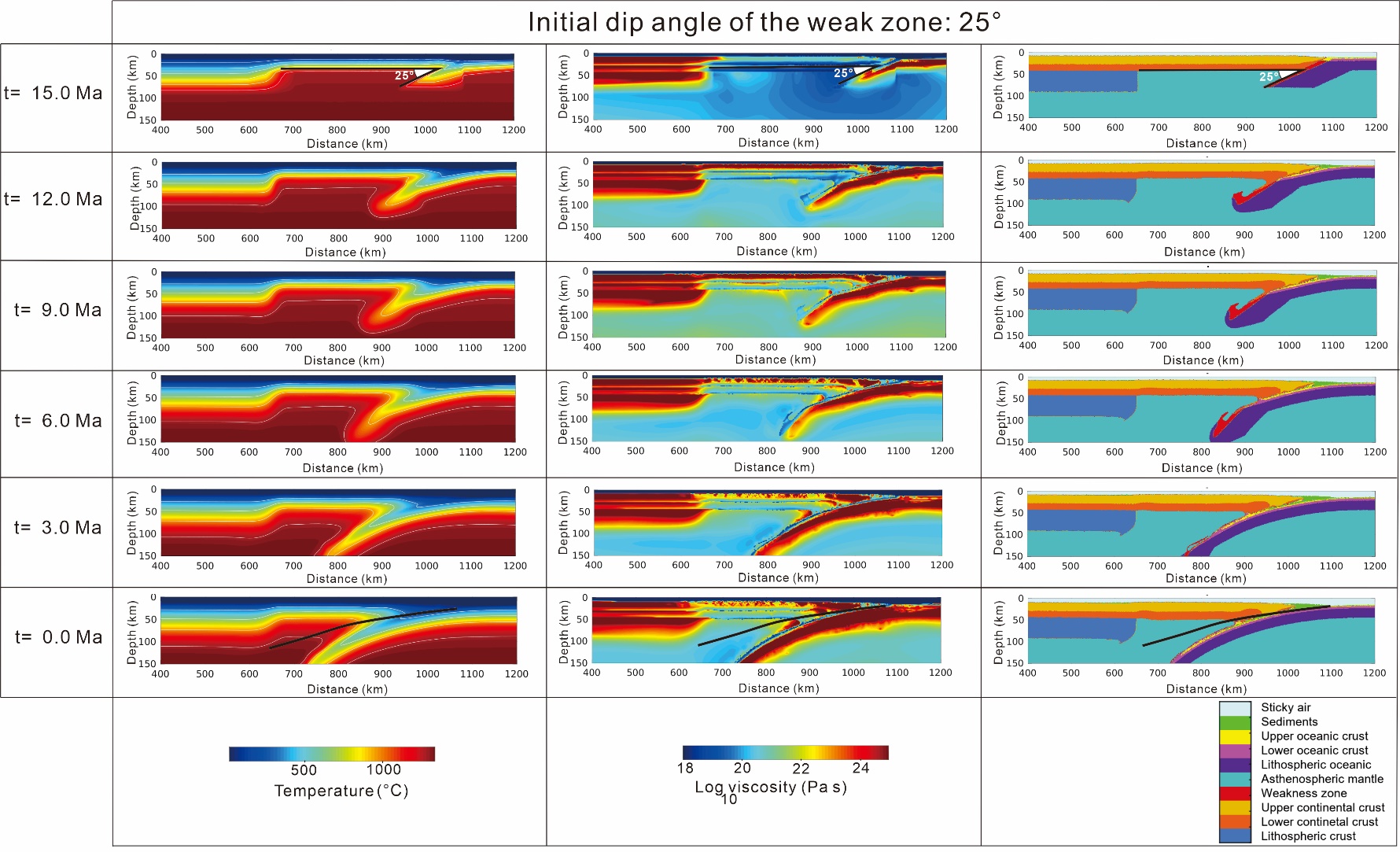
**

**Figure S4.** Time evolution showing results with a plate motion velocity and age imposed on the PHS plate based on convergence rate and age changes shown in Fig. 2A and initial dip of the weak zone of 25°. In this hypothesis, the PHS slab moves with rates close to zero between ~11- 7 Ma and initiates subduction with an approximate age plate of ~11 Ma^1^. Our models obtain steep subduction by imposing an initial dip angle of 25° for the weak zone. The black curve represents the slab geometry plotted over the final time step, and these values are obtained from https://www.mri-jma.go.jp/Dep/sei/fhirose/plate/en.Tools.html^3,4,5^.

**
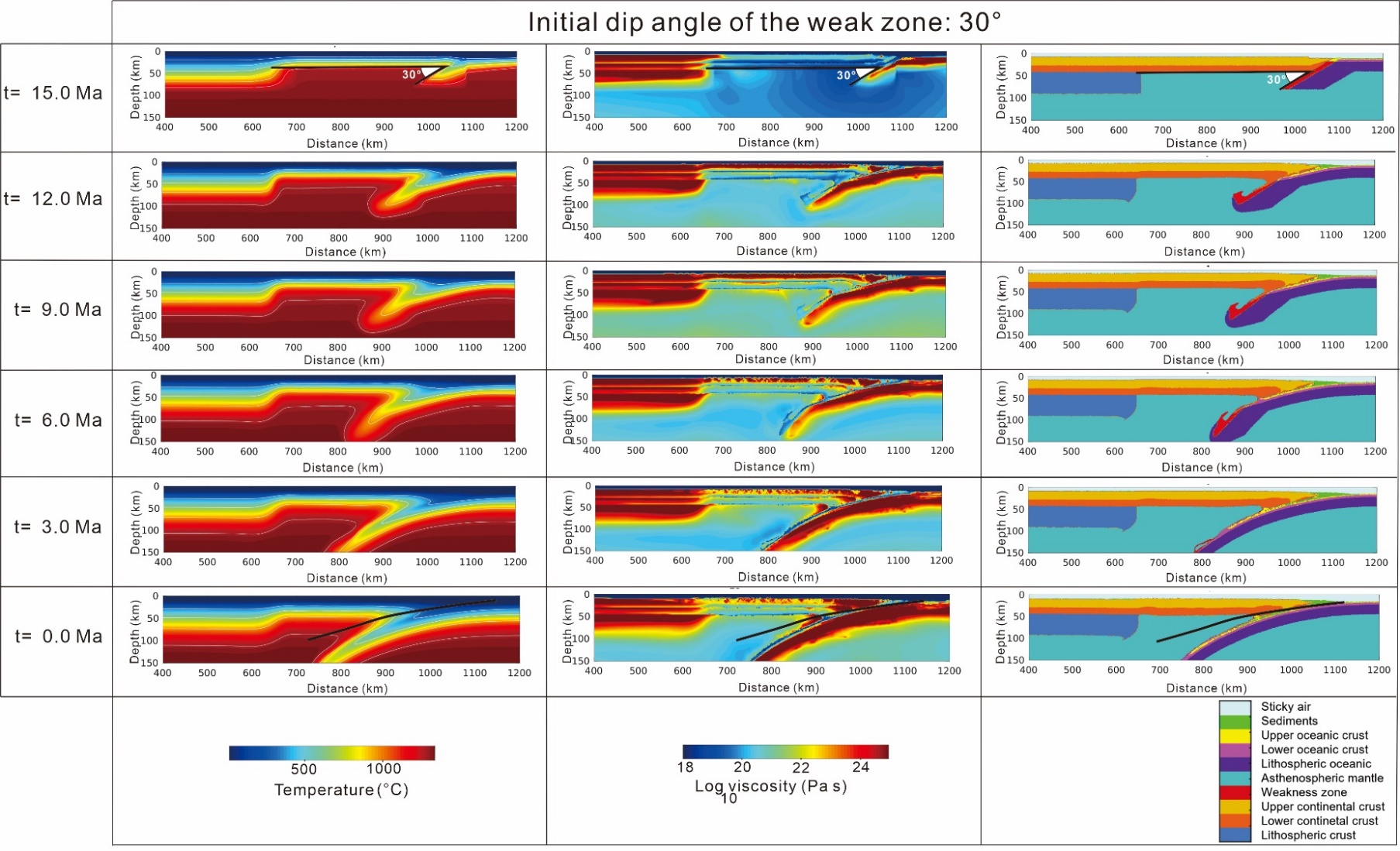
**

**Figure S5.** Time evolution showing results with a plate motion velocity and age imposed on the PHS plate based on convergence rate and age changes shown in Fig. 2A and initial dip of the weak zone of 30°. In this hypothesis, the PHS slab moves with rates close to zero between ~11- 7 Ma and initiates subduction with an approximate plate age of ~11 Ma^1^. Our models obtain steep subduction by imposing an initial dip angle of 30° for the weak zone. The black curve represents the slab geometry plotted over the final time step, and these values are obtained from https://www.mri-jma.go.jp/Dep/sei/fhirose/plate/en.Tools.html^3,4,5^.

**3.2 Simulations with plate motion velocity and age according to Model 2**

Supplementary Figs. S6, S7, S8, and S9 show time evolution with temperature, viscosity and composition with convergence rates and ages based on Fig. 2B, and initial dip angles of the weak zone between 15°-30°. This hypothesis is based on a high convergence rate of the PHS plate between 15 -3 Ma, changing its direction of motion from north-northeast to northwest after 3 Ma^2^. In this model, the plate initiates subduction with an age of approximately ~5.1 Ma at the Nankai Trough and increases over time^2^. Our numerical models obtained a good agreement with the observed current slab geometry along a profile passing through the Shikoku and Chugoku regions (Fig. 1) for initial dip angles of the weak zone between 20°-30° (Figs. S7, and S9). For dip angles of the weak zone of 15°, a slab geometry that reassembles flat subduction is obtained (Fig. S6).


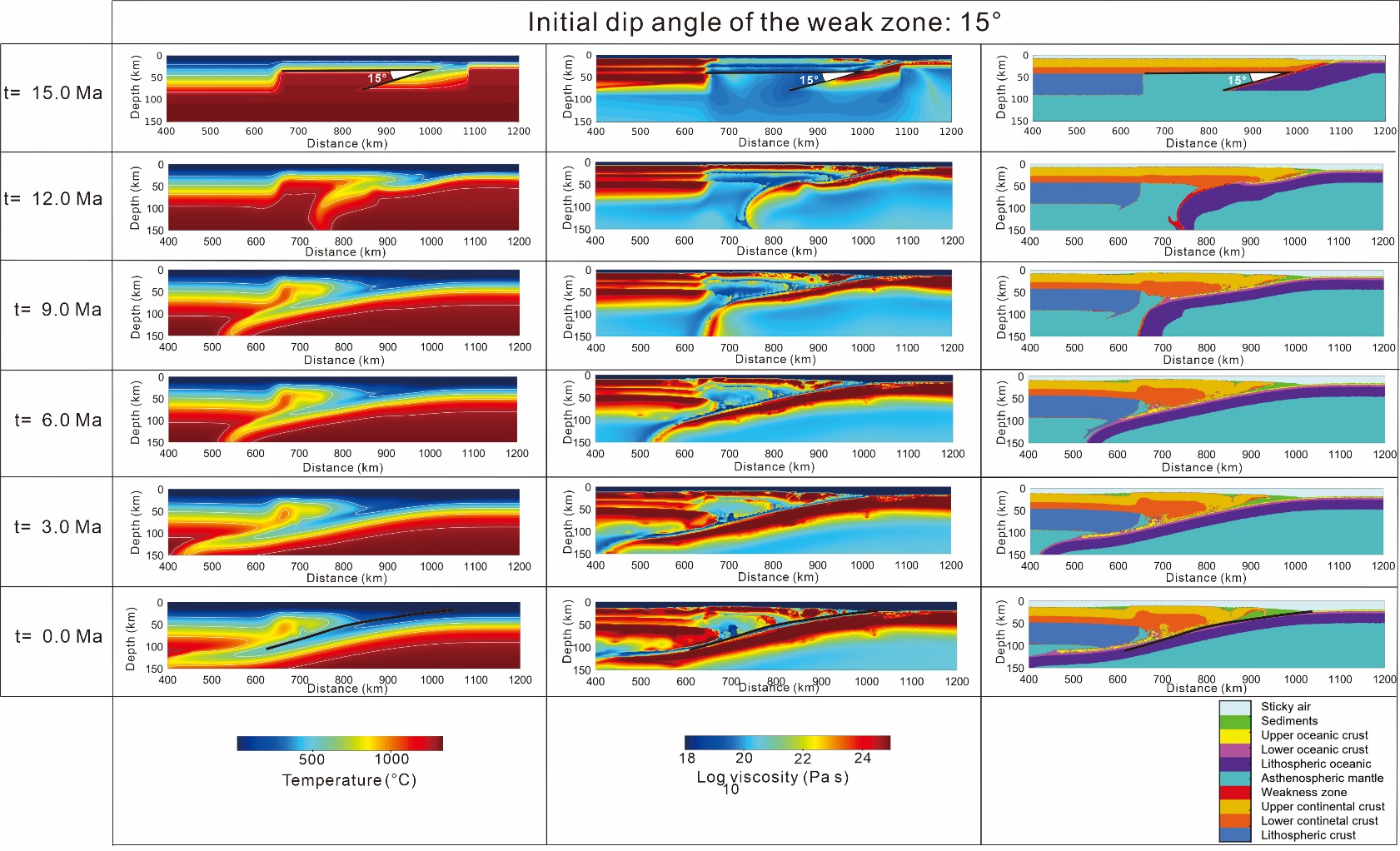


**Figure S6.** Time evolution showing results with a convergence rate and age imposed on the slab based on convergence rate and age changes shown in Fig. 2B^2^. The PHS slab moves with a high convergence rate between ~15-3 Ma, during this period it moves with a constant velocity of 7.33 cm/yr. At 3 Ma PHS plate reduces its convergence rate to ~5.6 cm/yr, and the normal convergence rate to the Nankai Trough remains constant from 3 Ma to the present^2^. Our models obtain flat subduction by imposing an initial dip angle of 15° for the weak zone. The black curve represents the slab geometry plotted over the final time step, and these values are obtained from https://www.mri-jma.go.jp/Dep/sei/fhirose/plate/en.Tools.html^3,4,5^

**
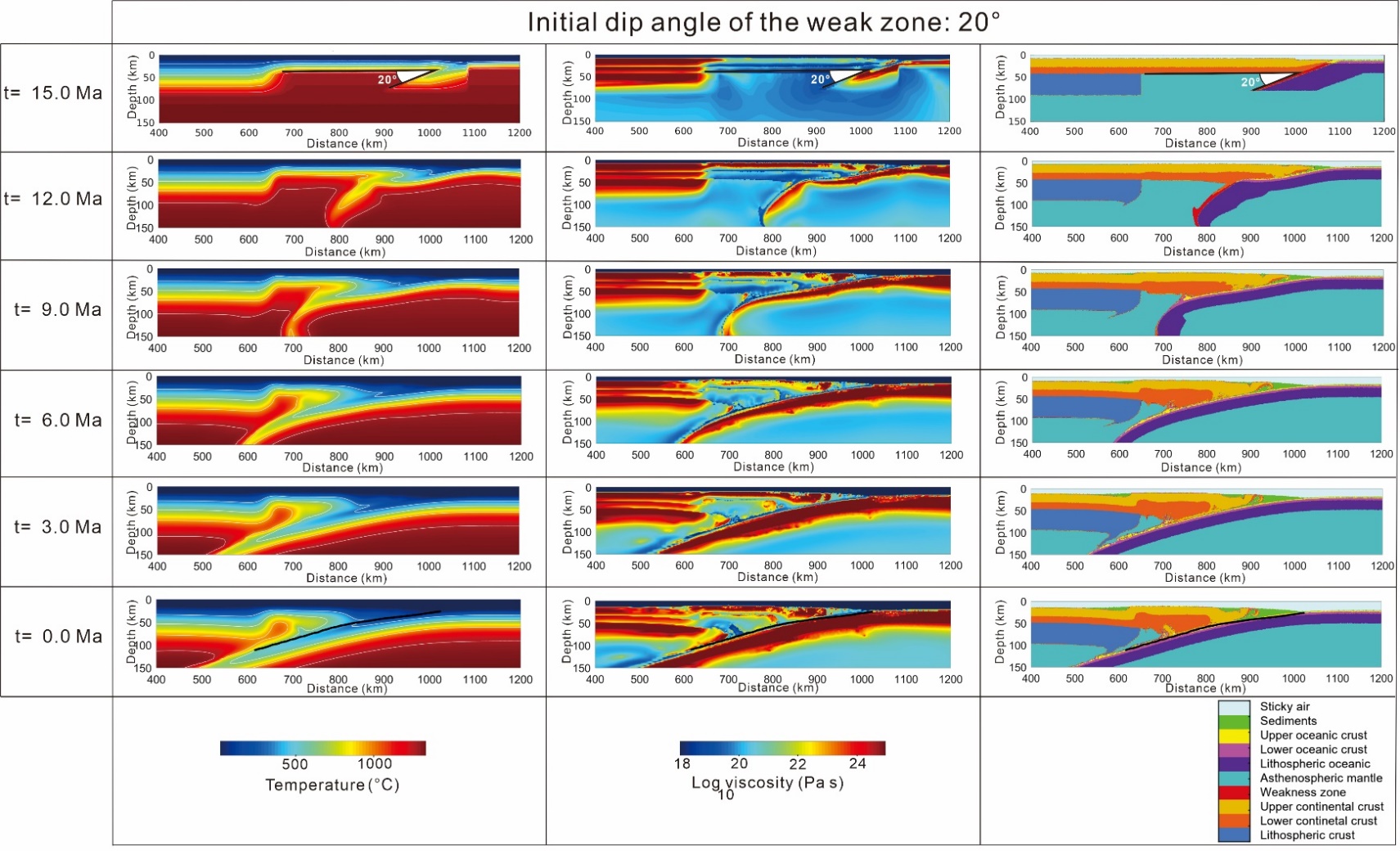
**

**Figure S7.** Time evolution showing results with a convergence rate and age imposed on the slab based on convergence rate and age changes shown in Fig. 2B^2^. The PHS slab moves with a high convergence rate between ~15-3 Ma, during this period it moves with a constant velocity of 7.33 cm/yr. At 3 Ma PHS plate reduces its convergence rate to ~5.6 cm/yr, and the normal convergence rate to the Nankai Trough remains constant from 3 Ma to the present^2^. The results are obtained by imposing an initial dip angle of 20° for the weak zone. The black curve represents the slab geometry plotted over the final time step, and these values are obtained from https://www.mri-jma.go.jp/Dep/sei/fhirose/plate/en.Tools.html^3,4,5^.

**
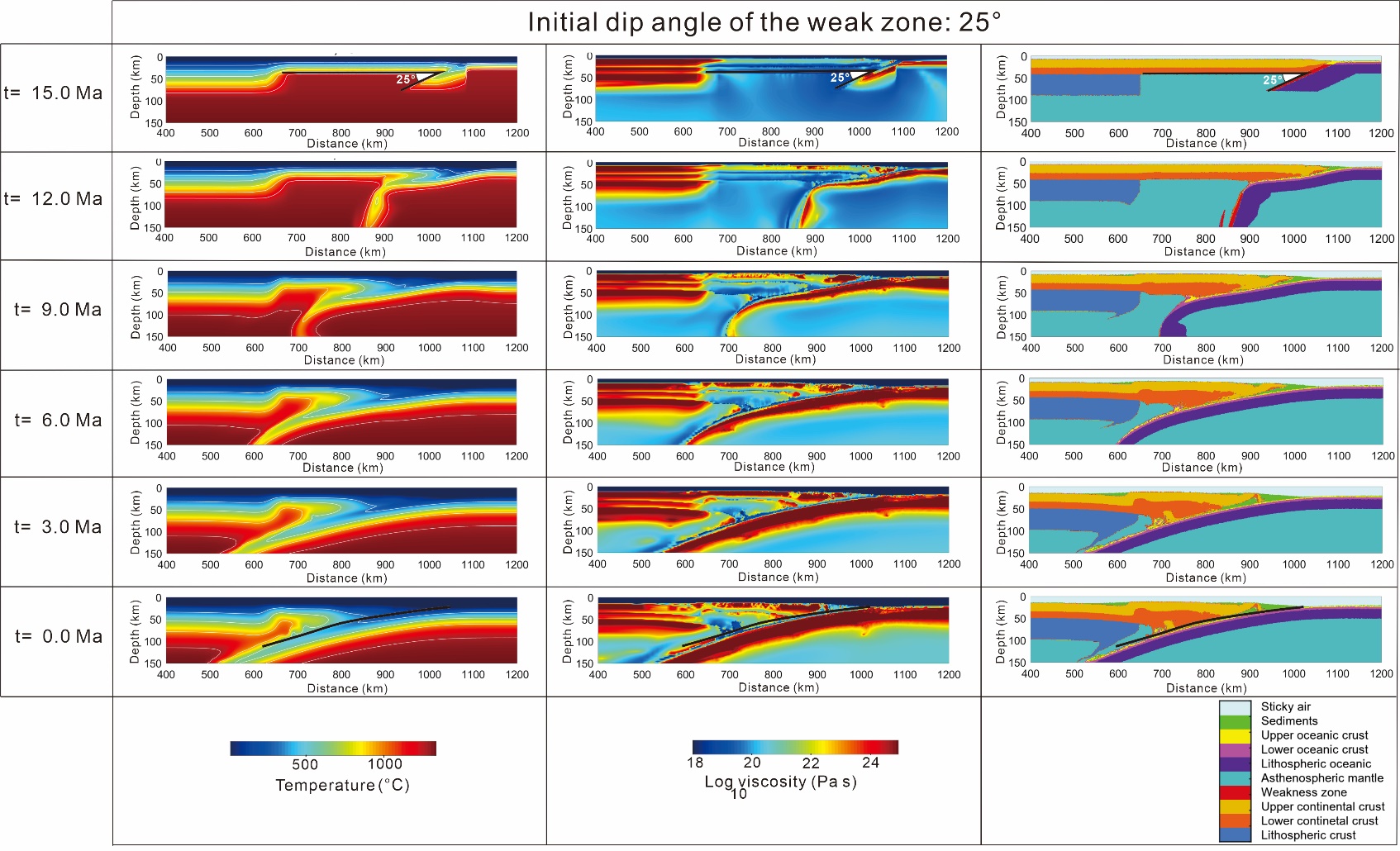
**

**Figure S8.** Time evolution showing results with a convergence rate and age imposed on the slab based on convergence rate and age changes shown in Fig. 2B. The PHS slab moves with a high convergence rate between ~15-3 Ma, during this period it moves with a constant velocity of 7.33 cm/yr. At 3 Ma PHS plate reduces its convergence rate to ~5.6 cm/yr, and the normal convergence rate to the Nankai Trough remains constant from 3 Ma to the present^2^. Our models obtain shallow subduction by imposing an initial dip angle of 25° for the weak zone. The black curve represents the slab geometry plotted over the final time step; these values are obtained from https://www.mri-jma.go.jp/Dep/sei/fhirose/plate/en.Tools.html^3,4,5^.

**
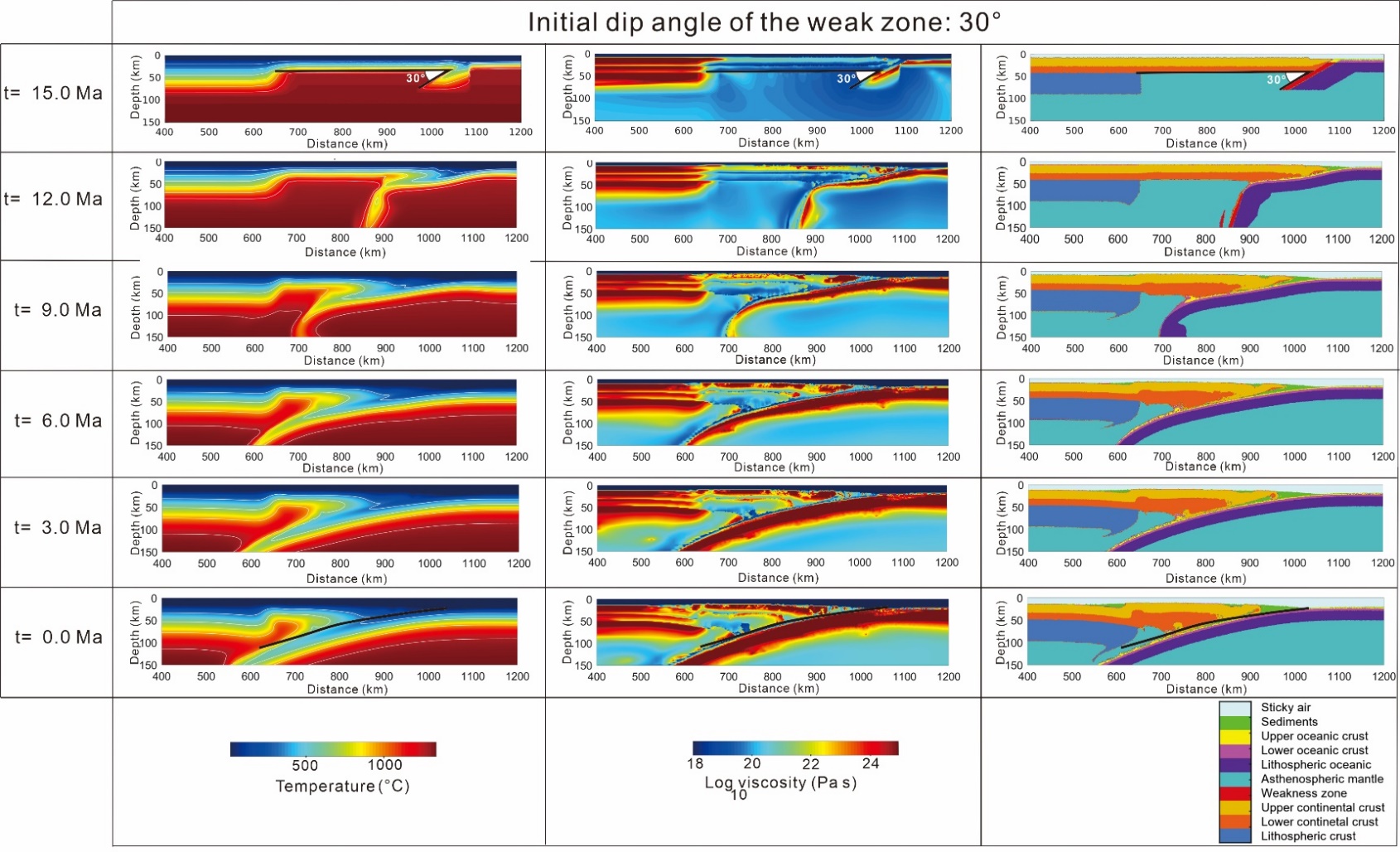
**

**Figure S9.** Time evolution showing results with a convergence rate and age imposed on the slab based on convergence rate and age changes shown in Fig. 2B. The PHS slab moves with a high convergence rate between ~15-3 Ma, during this period it moves with a constant velocity of 7.33 cm/yr. At 3 Ma PHS plate reduces its convergence rate to ~5.6 cm/yr, and the normal convergence rate to the Nankai Trough remains constant from 3 Ma to the present^2^. Our models obtain shallow subduction by imposing an initial dip angle of 30° for the weak zone. The black curve represents the slab geometry plotted over the final time step, and these values are obtained from https://www.mri-jma.go.jp/Dep/sei/fhirose/plate/en.Tools.html^3,4,5^.

**3.3 Sensitivity of the dip subduction angle to zones of high viscosity in the mantle**

Although seismic tomography shows that the PHS slab does not interact with the 410 km and 660 km transitions^5^ the cold viscous Pacific slab might locally affect mantle convection, flow, and the PHS slab dip angle^23,24,25,26^. All of the above models include the high viscosity below 500 km, which would be represented by the PAC slab^5,13,27^. Below we compare the above models with models that do not include a high-viscosity layer at the bottom of the model with models that include a high-viscosity layer.

Our models show that Model 2 shows great agreement with the slab geometry below the Shikoku and Chugoku regions^3,4,5^. When the high-viscosity layer is not included, the models evolve as a function of initial dip angle, age, and convergence rate. However, by including the high-viscosity and cold layer and expanding the computational domain in vertical direction, our models are relatively independent of the initial dip angle of the weak zone (in particular, for our preferred Model 2).

| **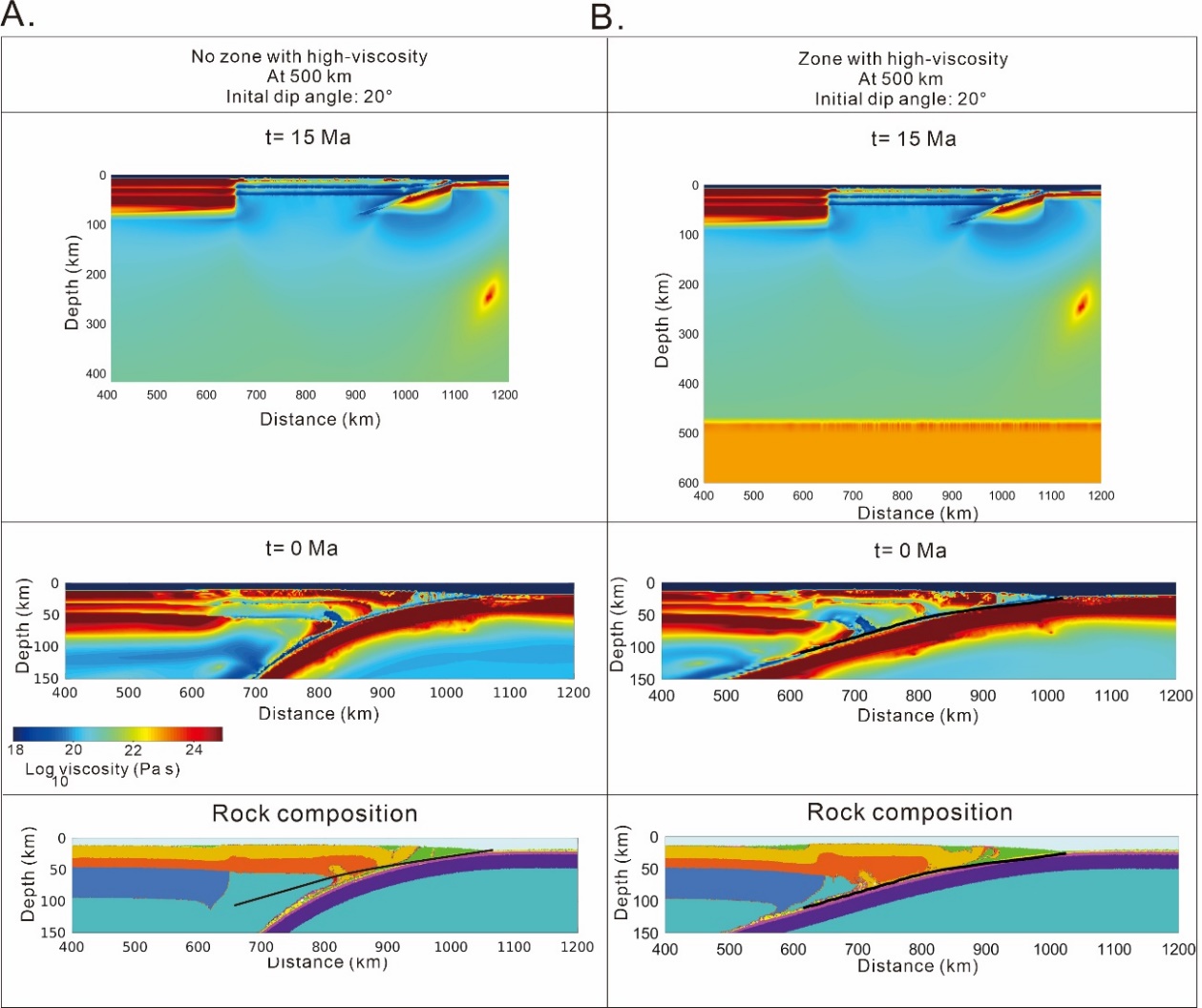** |
| --- |
| **Figure S10. A.** Model 2 without high-viscosity layer below 500 km. **B.** Model 2 with high-viscosity layer of $1\times{10}^{23}$ Pas with an approximate temperature of ~900°C below 500 km^5,13,27^ with a convergence rate and age imposed on the PHS plate based on convergence rate and age changes shown in Fig. 2B. The PHS slab moves with a high convergence rate between ~15-3 Ma, during this period it moves with a constant velocity of 7.33 cm/yr. At 3 Ma, the PHS plate reduces its convergence rate to ~5.6 cm/yr, and the normal convergence rate to the Nankai Trough remains constant from 3 Ma to the present^2^. Our models obtain steep subduction by imposing an initial dip angle of 20° for the weak zone. The black curve represents the slab geometry plotted over the final time step, and these values are obtained from https://www.mri-jma.go.jp/Dep/sei/fhirose/plate/en.Tools.html^3,4,5^. |

**3.4 Analysis of stress directions**

Model 1 shows extensional regime in the across-arc direction on the upper continental crust and in some areas of the lower continental crust. This behavior is maintained between 12-6 Ma as a consequence of the cessation of subduction. After 6 Ma, the stress directions change from extension to shortening for the upper continental crust. This is mainly determined by plate convergence. Between 6 Ma and the present, there is a smooth transition with almost equal extensional and shortening components at upper continental crust. However, the lower continental crust is dominated by shortening directions perpendicular to the trench. Model 2 shows similar characteristics.

The state of stress in SW Japan is still controversial because it is strongly related to the onset of subduction, the culmination of the opening of the Japan Sea and the clockwise rotation of SW Japan^1,2^. Previous studies suggested that the stress regime changed after 15 Ma from a predominantly extensional regime to a predominantly compressional regime with an orientation perpendicular to the arc^30^. On the other hand, studies suggest a stress change from north-south extensional to north-south compressional in SW Japan at 15 Ma^29^. This is a consequence of the opening of the Japan Sea^1,2^ and the onset of subduction along the Nankai Trough . The eruption of high-Mg andesites forming the Setouchi volcanic arc changes the compressional regime to an extensional regime in SW Japan between 14-12 Ma^29,30^. Between 12 Ma and the present, SW Japan is subjected to a strong compressional regime by the subduction of the PHS plate^29,30^

A.

| **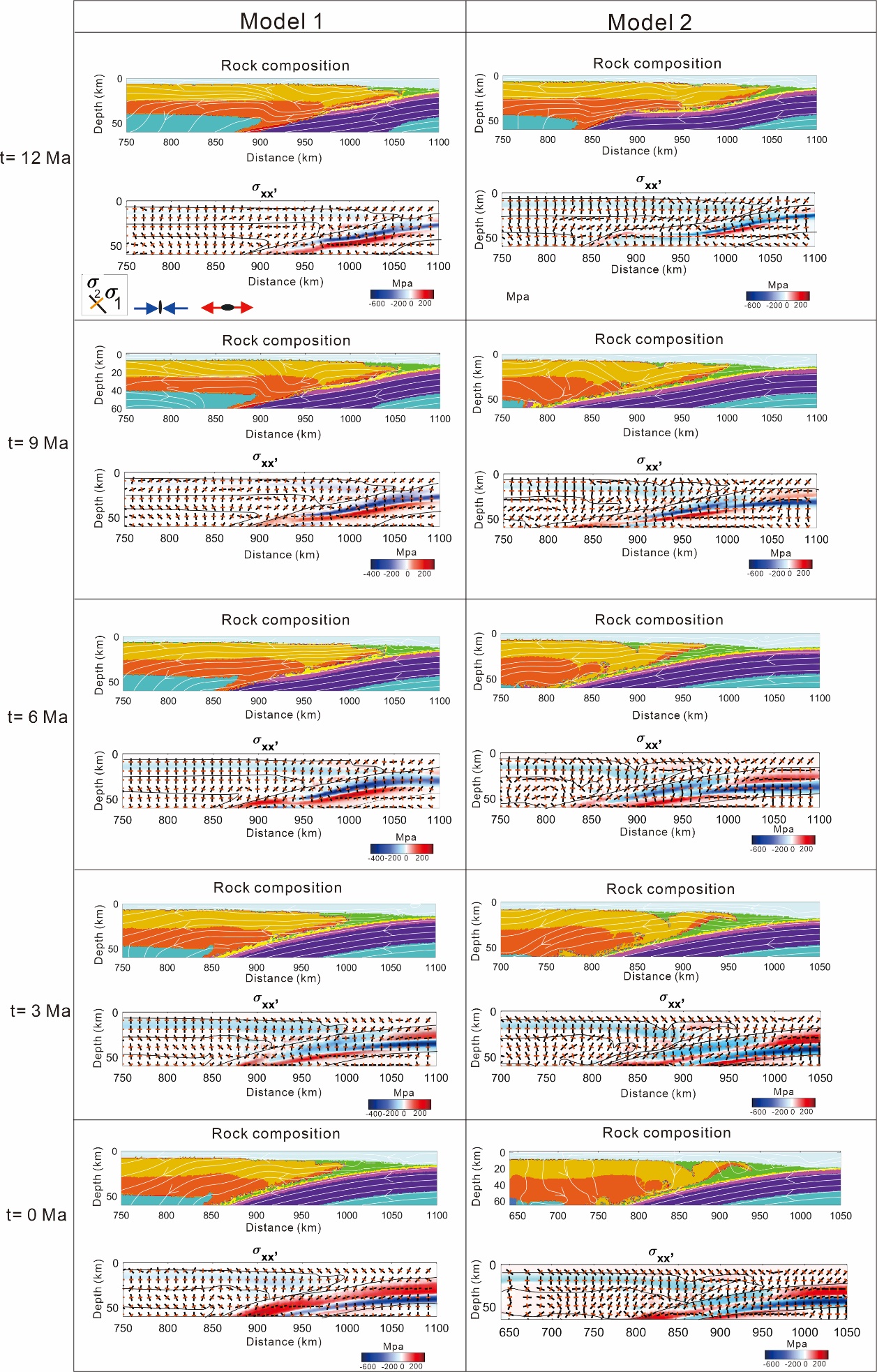**  B. |
| --- |
| **Figure S11. A.** Deviatoric stress with stress direction for Model 1. **B.** Deviatoric stress with stress direction for Model 2. Evolution from 12 Ma to the present of the normal component of deviatoric stress and stress orientation. The short oranges bars in the horizontal position represent shortening and the black bars in the horizontal position represent extension directions, respectively^7^. Between 12 -0 Ma, both models reflect high compression near the subduction zone. However, Model 2 experiences higher compression than Model 1 due to the high convergence rate. |

Our numerical models predict only strong compression in the upper continental crust, especially in Model 2 due to the high convergence rate, and present extensional components in the lower continental crust above a region where the slab increases the dip angle and sink into the mantle.

**3.5 Influence of slab viscosity on subduction style**

We investigated whether the changes in viscosity influence on the modeling results. We performed additional numerical models using different values of the cutoff viscosity upper limits. Compared with the numerical model presented in the manuscript, these additional models use an upper limit for the viscosity cutoff with one order of magnitude higher and lower that the reference value of 10^25^ Pa s (Fig. S11B). Namely, we use an upper limit of the viscosity cutoff of $1\times{10}^{24}$ Pa s (Fig. S11A) and 1 x 10^26^ Pa s (Fig. S11C), respectively. Both models are performed for the model setup with an initial dip angle of the weak zone of 20°. Modeling results show that the main conclusion is not altered, and the hypothesis of Tatsumi et al. (2020)^2^ (Model 2) produced a shallower slab geometry than Kimura et al. (2014) ^1^ Model 1.

| 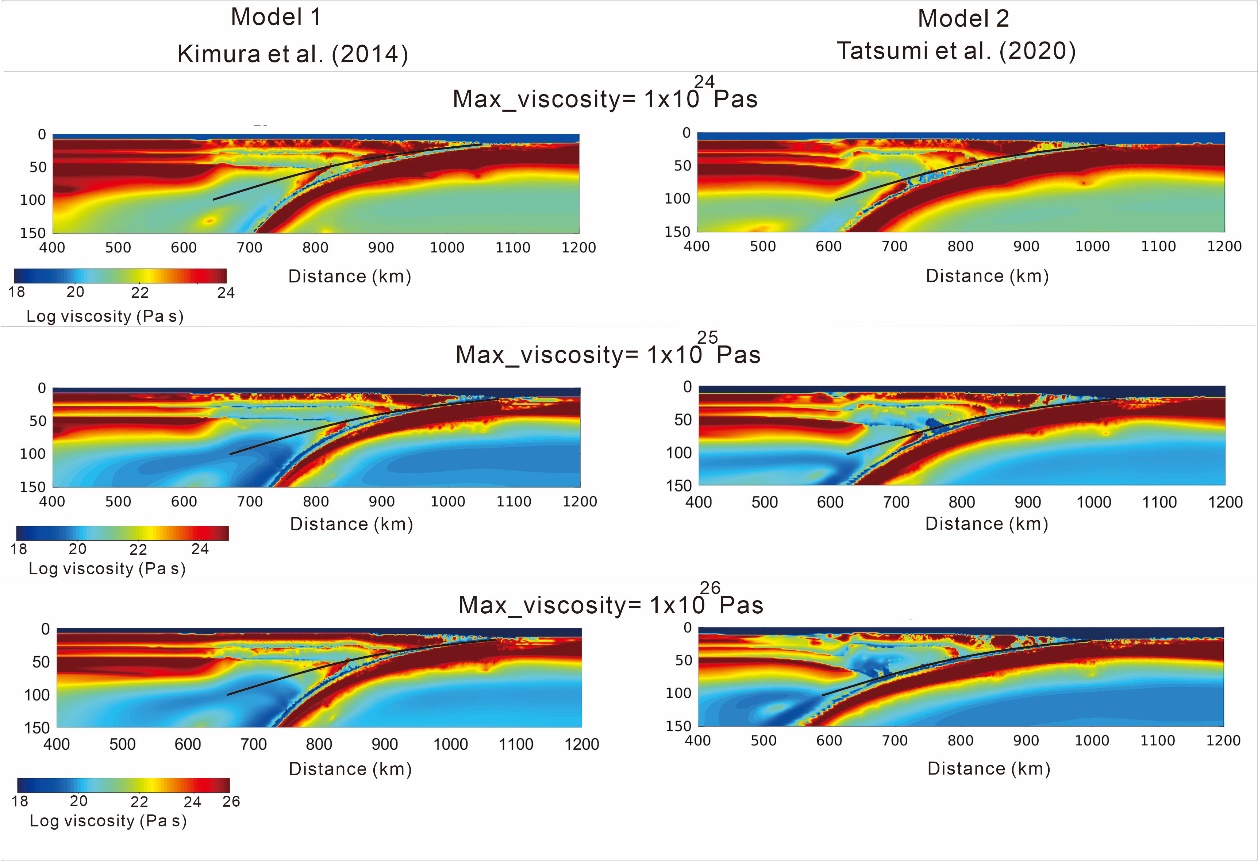 |
| --- |
| **Figure S12.** Comparison between Model 1 and Model 2 using three values of viscosity cutoff: $1\times{10}^{24}$ Pa s, $1\times{10}^{25} Pa s$and 1 x 10^26^ Pa s, respectively with initial dip angle of the weak zone of 20°. **A**. Model 1 with plate motion velocity and age conditions of Fig. 2A. **B** Model 2 with plate motion velocity and age conditions of Fig. 2B |

**3.6 Influence of weak zone rheological properties on the onset of subduction.**

Another parameter we tested was the viscosity of the initial weak zone that helps the two plates to decouple in the initial stage of subduction (subduction initiation). Without the weak zone, the two plates do not decouple, and subduction never occurs. We investigate the influence of the weak zone on the modeling results using plasticity conditions. Plasticity conditions are defined in terms of material cohesion, internal friction angle and dynamic pressure^6,7^. At the onset of subduction, the boundary between the continental and oceanic plates undergoes large compressions, resulting in a decrease in viscosity due to the power-law ductile creep^6^. The models presented in the main body of our manuscript evolve under cohesion and internal friction angle conditions of c=10^6^ Pa (or 1 MPa)$\mathrm{and} \sin\left( \right)$=0.03. We observed that increasing the weak zone cohesion with two orders of magnitude (i.e., 100 MPa) and internal friction angle to $\sin\left( \right)$**=**0.05, the viscosity in the weak zone does not decrease and in this case large deformations and no decoupling at the contact area between the two plates (see Fig. S13A). On the other hand, if we reduce the weak zone cohesion and internal friction (i.e., c=10^4^ Pa, $\sin\left( \right)$=0.01), the two plates successfully decouple, and we obtain a realistic subduction zone (Fig. S13 B).

| **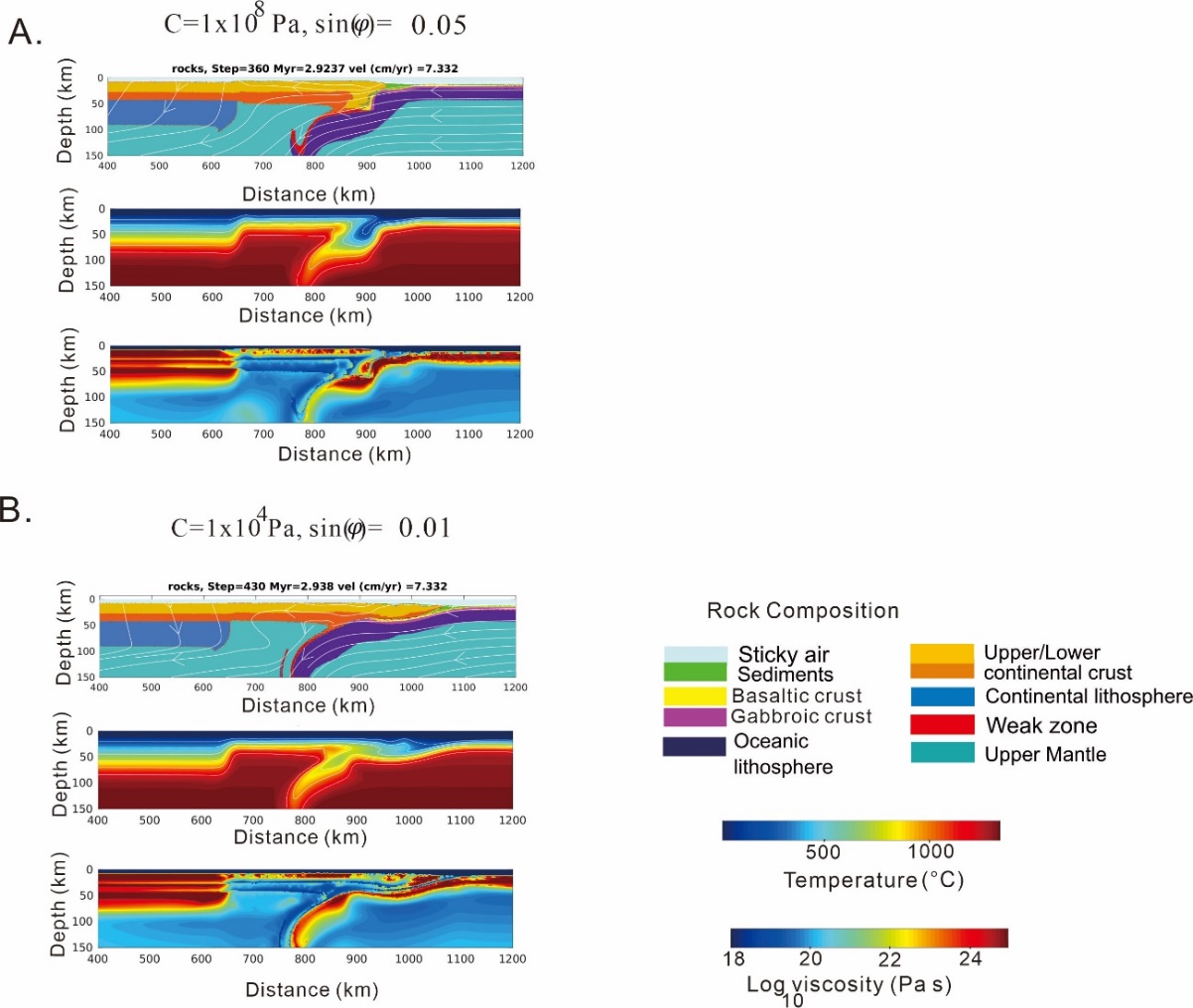** |
| --- |
| **Figure S13.** Model 1 reproduced with initial dip angle of 15° with cohesion conditions and internal friction angle of **A**. c=10^8^ Pa and $\sin\left( \right)$**=**0.05. **B.** c=10^4^ Pa and $\sin\left( \right)$**=**0.01. |

**4. Supplementary Animations**

**Supplementary animation 1**

This animation shows the temperature and rock composition for the PHS slab. This model is reproduced under an initial dip angle for the weak zone of 15° and plate motion velocity and age conditions of Fig. 2A. The model evolves over a period of 15 Myr and exhibits steep subduction.

**Supplementary animation 2**

This animation shows the temperature and rock composition for the PHS slab. This model is reproduced under an initial dip angle for the weak zone of 15° and plate motion velocity and age conditions of Fig. 2B. The model evolves over a period of 15 Myr and exhibits shallow subduction.

**5. Supplementary References**

1. Kimura, G., Hashimoto, Y., Kitamura, Y., Yamaguchi, A., & Koge, H. Middle Miocene swift migration of the TTT triple junction and rapid crustal growth in southwest Japan: A review. *Tectonics*. **33**, 1219-1238 (2014).
2. Tatsumi, Y., Suenaga, N., Yoshioka, S., Kaneko, K., & Matsumoto, T. Contrasting volcano spacing along SW Japan arc caused by difference in age of subducting lithosphere. *Scientific Reports.* **10(1)**, 15005 (2020).
3. Hirose, F., Nakajima, J., & Hasegawa, A. Three‐dimensional seismic velocity structure and configuration of the Philippine Sea slab in southwestern Japan estimated by double‐difference tomography. *J. Geophys. Res. Solid Earth*. **113** (2008).
4. Baba, T., Y. Tanioka, P. R. Cummins & Uhira K., The slip distribution of the 1946 Nankai earthquake estimated from tsunami inversion using a new plate model, *Phys. Earth Planet. Inter*. **132**, 59-73 (2002).
5. Nakajima, J., & Hasegawa, A. Subduction of the Philippine Sea plate beneath southwestern Japan: Slab geometry and its relationship to arc magmatism. *J. Geophys. Res. Solid Earth.* **112** (2007).
6. Gerya, T. Introduction to numerical geodynamic modelling. Cambridge University Press (2019).
7. Gerya, T. V., & Yuen, D. A. Robust characteristics method for modelling multiphase visco-elasto-plastic thermo-mechanical problems. *Physics of the Earth and Planetary Interiors.* **163**, 83-105 (2007).
8. Ranalli, G. Rheology of the Earth. *Springer Science & Business Media*. (1995).
9. Behr, W. M., Holt, A. F., Becker, T. W., & Faccenna, C. The effects of plate interface rheology on subduction kinematics and dynamics. *Geophys. J. Int*. **230**, 796-812 (2022).
10. Huangfu, P., Wang, Y., Cawood, P. A., Li, Z. H., Fan, W., & Gerya, T. V. Thermo-mechanical controls of flat subduction: Insights from numerical modeling. *Gondwana Research*. **40,** 170-183 (2016).
11. Burkett, E. R., & Billen, M. I. Three‐dimensionality of slab detachment due to ridge‐trench collision: Laterally simultaneous boudinage versus tear propagation. *Geochem. Geophys. Geosy*s. **11** (2010).
12. Turcotte D., & Schubert, G. *Geodynamics* (Third). Cambridge University Press (2014).
13. Wu, J., Suppe, J., Lu, R., & Kanda, R. Philippine Sea and East Asian plate tectonics since 52 Ma constrained by new subducted slab reconstruction methods. *Journal of Geophysical Research: Solid Earth*. **121(6)**, 4670-4741 (2016).
14. Lallemand, S., Font, Y., Bijwaard, H., & Kao, H. New insights on 3-D plates interaction near Taiwan from tomography and tectonic implications. *Tectonophysics*. **335(3-4)**, 229-253 (2001).
15. Yoshioka, S., Suminokura, Y., Matsumoto, T., & Nakajima, J. Two-dimensional thermal modeling of subduction of the Philippine Sea plate beneath southwest Japan. *Tectonophysics.* **608**, 1094-1108 (2013).
16. Suenaga, N., Yoshioka, S., & Matsumoto, T. Relationships among temperature, dehydration of the subducting Philippine Sea plate, and the occurrence of a megathrust earthquake, low-frequency earthquakes, and a slow slip event in the Tokai district, central Japan. *Physics of the Earth and Planetary Interiors*. **260**, 44-52 (2016).
17. Shiono, K. Seismicity of the SW Japan arc-subduction of the young Shikoku basin. *Modern Geology.* **12**, 449-464 (1988).
18. Okino, K., Shimakawa, Y., & Nagaoka, S. Evolution of the Shikoku basin. *Journal of geomagnetism and geoelectricity.* **46(6)**, 463-479 (1994).
19. Hoshi, H., Kato, D., Ando, Y., & Nakashima, K. Timing of clockwise rotation of Southwest Japan: constraints from new middle Miocene paleomagnetic results. *Earth, Planets and Space*. **67(1)**, 1-13 (2015).
20. Sella, G. F., Dixon, T. H., & Mao, A. REVEL: A model for recent plate velocities from space geodesy. *Journal of Geophysical Research: Solid Earth.* **107(B4)**, ETG-11 (2002).
21. Takahashi, M., Tectonic development of the Japanese Islands controlled by Philippine Sea Plate motion. *Journal of Geography (Chigaku Zasshi)*.**115(1)**, 116-123 (2006).
22. Demets, C., Gordon, R., & Argus, D. Geologically current plate motions. *Geophysical journal international.* **1**, 1-80 (2010).
23. Holt, A. F., Royden, L. H., Becker, T. W., & Faccenna, C. Slab interactions in 3-D subduction settings: The Philippine Sea Plate region. *Earth and Planetary Science Letters*. **489**, 72-83 (2018).
24. Faccenna, C., Holt, A. F., Becker, T. W., Lallemand, S., & Royden, L. H. Dynamics of the Ryukyu/Izu-Bonin-Marianas double subduction system. *Tectonophysics*. **746,** 229-238 (2018).
25. Balázs, A., Faccenna, C., Ueda, K., Funiciello, F., Boutoux, A., Blanc, E. J. P., & Gerya, T. Oblique subduction and mantle flow control on upper plate deformation: 3D geodynamic modeling. *Earth and Planetary Science Letters*. **569**, 117056 (2021).
26. Balazs, A., Faccena, C., Gerya, T. V., Ueda, K., & Funiciello, F. The dynamics of forearc-back-arc vertical motion: numerical models and observations from the Mediterranean. *Authorea Preprints* (2022).
27. Ma, J., Tian, Y., Zhao, D., Liu, C., & Liu, T. Mantle dynamics of western Pacific and East Asia: New insights from P wave anisotropic tomography. *Geochemistry, Geophysics, Geosystems*. **20(7)**, 3628-3658 (2019).
28. Yamamoto, H. Submarine geology and post-opening tectonic movements in the southern region of the Sea of Japan. *Marine geology*. **112 (1-4)**, 133-150 (1993).
29. Tatsumi, Y., Ishikawa, N., Anno, K., Ishizaka, K., & Itaya, T. Tectonic setting of high-Mg andesite magmatism in the SW Japan arc: K–Ar chronology of the Setouchi volcanic belt. *Geophysical Journal International*. **144 (3)**, 625-631 (2001).
30. Haji, T., Yamaji, A., Iwano, H., Danhara, T., & Hirata, T. Extensional stress accompanied by Miocene near-trench magmatism in the southern Kii Peninsula, SW Japan. *Journal of Asian Earth Sciences*. **235**, 105266 (2022).
